# GTSE1 promotes nasopharyngeal carcinoma proliferation and angiogenesis by upregulating STMN1

**DOI:** 10.1186/s13008-024-00119-9

**Published:** 2024-05-02

**Authors:** Jiadi Dong, Jingjing Chen, Yidong Wu, Jiangyu Yan

**Affiliations:** Department of Otorhinolaryngology Head and Neck Surger, Ningbo Medical Center Lihuili Hospital, No. 57, Xingning, Yinzhou, 315000 Zhejiang China

**Keywords:** Nasopharyngeal carcinoma, GTSE1, FOXM1, STMN1, Invasion, Angiogenesis

## Abstract

**Background:**

Nasopharyngeal carcinoma (NPC) is a malignant tumor with poor survival rate. G2 and S phase-expressed‐1 (GTSE1) takes part in the progression of diverse tumors as an oncogene, but its role and potential mechanism in NPC remain unknown.

**Methods:**

The GTSE1 expression was analyzed by western blot in NPC tissues and cells. Knock-down experiments were conducted to determine the function of GTSE1 in NPC by cell counting kit-8, the 5-ethynyl-2′-deoxyuridine (EdU) incorporation experiment, cell scratch wound-healing experiment, transwell assays, tube forming experiment and western blot. In addition, the in vivo role of GTSE1 was addressed in tumor-bearing mice.

**Results:**

The expression of was increased in NPC. Silencing of GTSE1 suppressed cell viability, the percent of EdU positive cells, and the number of invasion cells and tubes, but enhanced the scratch ratio in NPC cells. Mechanically, downregulation of GTSE1 decreased the expressions of FOXM1 and STMN1, which were restored with the upregulation of FOXM1. Increased expression of STMN1 reversed the effects of the GTSE1 silencing on proliferation, migration, invasion and angiogenesis of NPC cells. Furthermore, knockdown of GTSE1 repressed the tumor volume and tumor weight of xenografted mice.

**Conclusion:**

GTSE1 was highly expressed in NPC, and silencing of GTSE1 ameliorated the malignant processes of NPC cells by upregulating STMN1, suggesting a possible therapeutical target for NPC.

**Supplementary Information:**

The online version contains supplementary material available at 10.1186/s13008-024-00119-9.

## Introduction

Nasopharyngeal carcinoma (NPC) originates from the nasopharynx mucosal epithelium, is characterized with early lymphatic spread, rapid local invasion and distant metastasis [1, 2]. 96,371 new cases and 58,094 deaths of NPC are reported in 2020 around the world, with more than 70% of new cases occurring in Southeast and East Asia [3, 4]. Several factors, such as environmental influences, Epstein Barr virus (EBV) infection, dietary habits and genetic susceptibility, have been demonstrated to drive the progression and development of NPC [1]. However, its pathogenesis still needs to be further elucidated. The 10-year survival rate of patients with NPC diagnosed at the early stage has been improved through the radiotherapy and chemotherapy, standard treatment paradigms for NPC [5, 6]. However, most patients (more than 70%) are diagnosed in the advanced stage because NPC patients at the early stage often exhibit asymptoms or atypical symptoms. Thus, despite the advance on these therapies, the survival rate of NPC patients remains poor. Moreover, toxicity and side effects of radiotherapy and chemotherapy are huge obstacles to the quality of life of NPC patients [7]. Therefore, identifying new therapeutic targets is critical for the development of the diagnosis and treatment of NPC.

G2 and S phase-expressed‐1 (GTSE1), located on chromosome 22q13.2‐q13.3, is a microtubule‐localized protein, expressed specially during the cell cycle S and G2 phases [8, 9]. GTSE1 is regulated by P53, and in turn negatively modulates the P53 activity via binding to its C‐terminal regulatory domain, thereby decreasing apoptosis in a P53‐dependent fashion [10–12]. Thus, GTSE1 has been revealed to be closely related to the process of different tumors. GTSE1 has been revealed as a biomarker for the immunosuppressive tumor microenvironment based on a pan-cancer analyses [13], and high expression of GTSE1 is associated with poor patient survival in many cancer types, such as bladder cancer [14], acral melanoma [15], hepatocellular carcinoma [16, 17], lung cancer [18], clear cell renal cell carcinoma [19, 20], non-small-cell lung cancer [21], cervical cancer [22], and breast cancer [23]. Moreover, GTSE1 is identified to participate in the proliferation, migration, and invasion of bladder cancer [14]. acral melanoma [15], hepatocellular carcinoma [17], lung cancer [18], clear cell renal cell carcinoma [20], and non-small-cell lung cancer [21]. The role of GTSE1 in drug resistance is shown in gastric cancer cells [24], osteosarcoma [25], breast cancer [23], clear cell renal cell carcinoma [19], and non-small-cell lung cancer [26]. In addition, GTSE1 is demonstrated to be involved in apoptosis in clear cell renal cell carcinoma [20], gastric cancer cells [24] and esophageal squamous cell carcinoma [27], and it was also involved in Warburg effect in cervical cancer [28].Furthermore, GTSE1 has been identified to be upregulated in head and neck squamous cell carcinoma (HNSC) [13], and GTSE1 can act as one of nine genes contributing to build the model for the prognostic risk prediction of HNSC [29], which indicated that GTSE1 might be involved in the progression of NPC.

Thus, to address whether GTSE1 was consistently highly expressed in NPC and whether it is involved in the malignant process of NPC, such as proliferation, migration, invasion and angiogenesis, in vitro and in vivo experiments were conducted in the present study. The findings demonstrated that NPC cells expressed high levels of GTSE1, and GTSE1 knockdown suppressed proliferation, mobility, invasion and angiogenesis of NPC cells by upregulating STMN1. This is the first time to uncover the role of GTSE1 in NPC, providing a potential therapeutic target for NPC.

## Results

### GTSE1 was strongly expressed in NPC

**Fig. 1:**
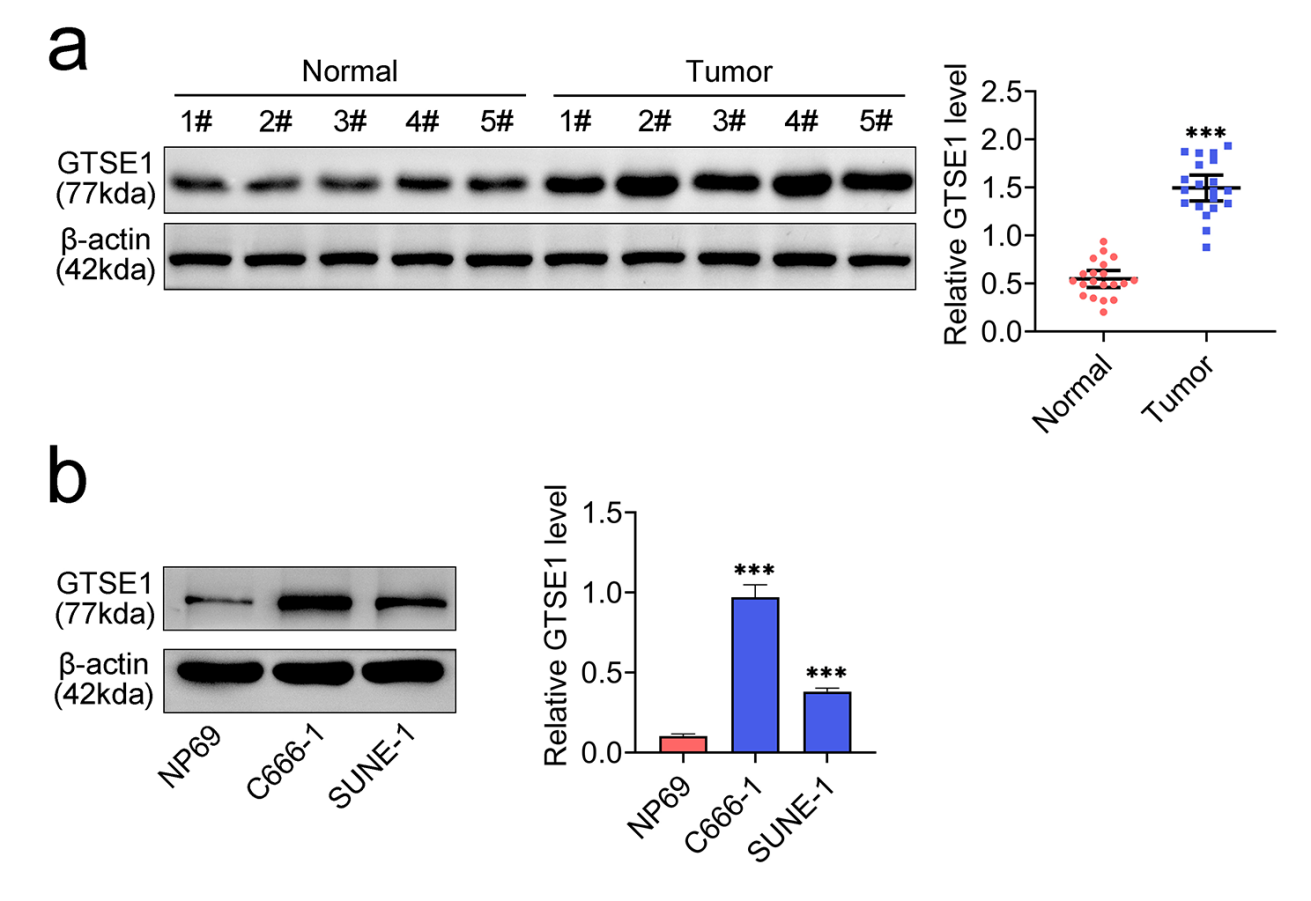
GTSE1 was highly expressed in NPC. **(a)** The relative protein expression of GTSE1 in tumor tissues from NPC patients was detected by western blot. Data were normalized with β-actin. **(b)** The relative protein expression of GTSE1 in NPC cell lines was detected by western blot. Data were normalized with β-actin. ***p < 0.001 vs. NP69

According to an analysis using tumor tissues from NPC patients, the expression of GTSE1 was considerably upregulated in NPC samples when compared to control samples (Fig. 1a). Also, the relative protein expression of GTSE1 was consistently and noticeably increased (Fig. 1b) in NPC cell lines (C666-1 and SUNE-1 cells). Thus, the level of GTSE1 was increased in NPC.

### GTSE1 knockdown slowed the growth of NPCs

**Fig. 2:**
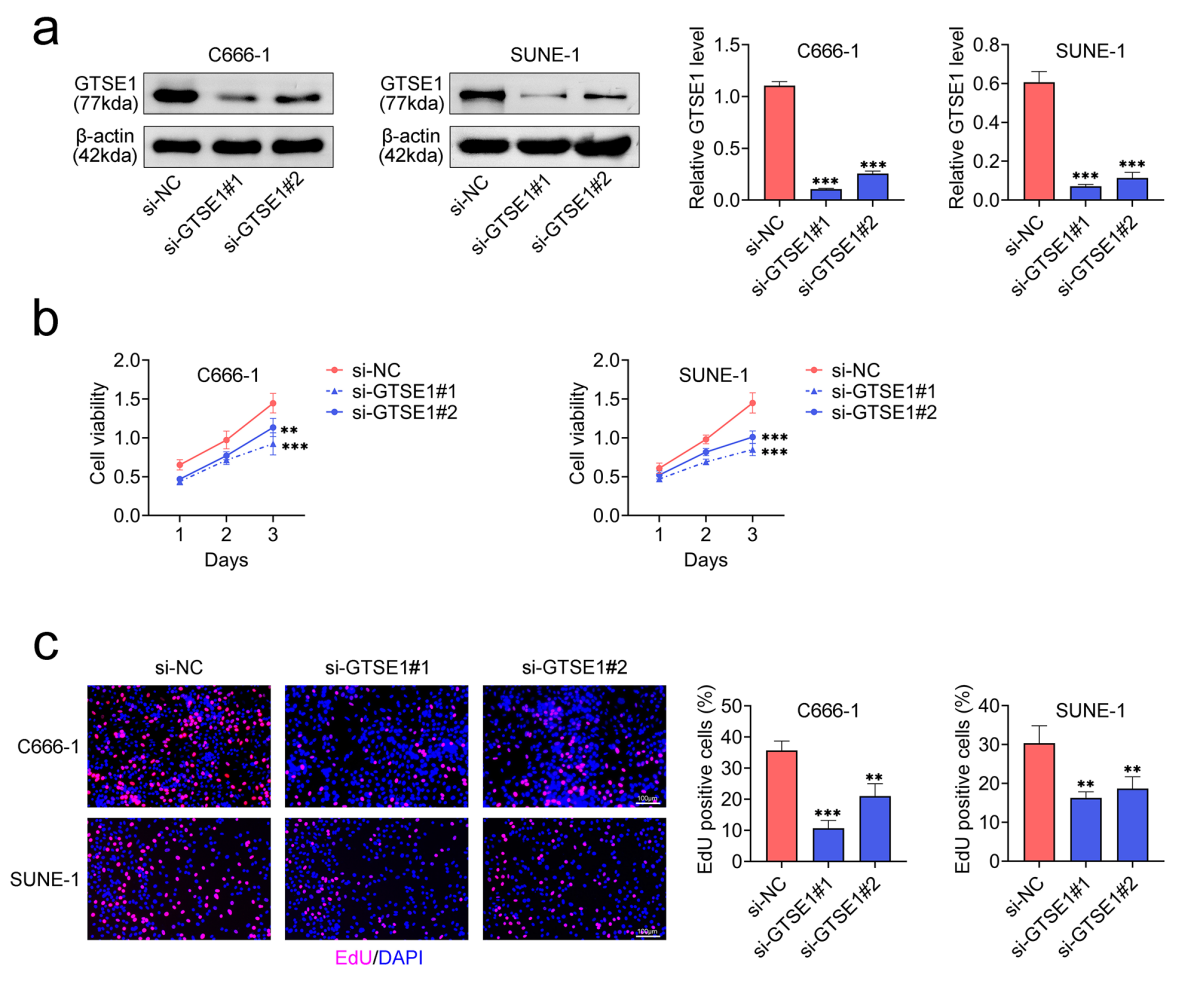
Knockdown of GTSE1 suppressed the NPC proliferation. Two siRNAs targeting GTSE1 (si-GTSE1#1 and si-GTSE1#2) were transfected into C666-1 and SUNE-1 cells to downregulate the level of GTSE1. **(a)** The relative protein expression of GTSE1 was detected by western blot. Data were normalized with β-actin. **(b)** Examination of cell viability by CCK-8. **(c)** Measurement of the percent of EdU positive cells by Edu staining. Scale bar = 100 μm. **p < 0.01 and ***p < 0.001 vs. si-NC

Two siRNAs targeting GTSE1 (si-GTSE1#1 and si-GTSE1#2) were transfected into C666-1 and SUNE-1 cells to downregulate the level of GTSE1 in order to investigate the role of GTSE1 in the progression of NPC. Both siRNAs targeting GTSE1 markedly decreased the relative protein expression of GTSE1 in C666-1 and SUNE-1 cells (Fig. 2a). Two siRNAs targeting GTSE1 transfected into C666-1 and SUNE-1 cells drastically reduced the number of EdU positive cells and the cell viability (Fig. 2b and c). Therefore, downregulation of GTSE1 suppressed proliferation of NPCs.

### Silencing of GTSE1 attenuated migration, invasion and angiogenesis of NPC cells

**Fig. 3:**
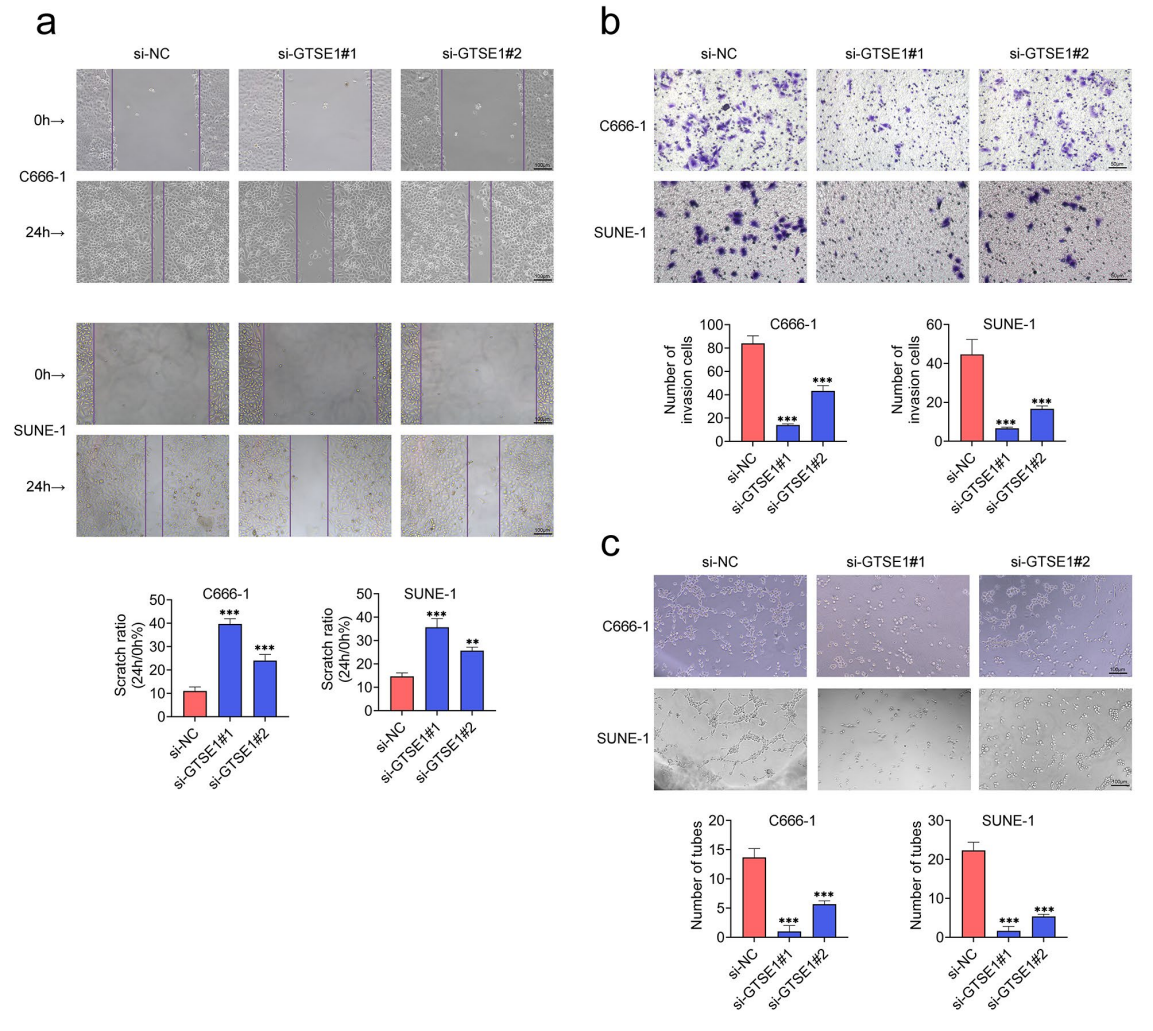
Knockdown of GTSE1 reduced migration, invasion and angiogenesis of NPC cells. Two siRNAs targeting GTSE1 (si-GTSE1#1 and si-GTSE1#2) were transfected into C666-1 and SUNE-1 cells to downregulate the level of GTSE1. **(a)** The migration ability was assessed by the cell scratch wound-healing experiment. Scale bar = 100 μm. **(b)** The invasion ability was evaluated by transwell assays. Scale bar = 50 μm. **(c)** The angiogenesis ability was determined by the tube forming experiment. Scale bar = 100 μm. **p < 0.01 and ***p < 0.001 vs. si-NC

Then, C666-1 and SUNE-1 cells were transfected with si-GTSE1#1 and si-GTSE1#2 to address the role of GTSE1 in migration, invasion and angiogenesis. Transfection of both two siRNAs targeting GTSE1 into C666-1 and SUNE-1 cells caused a prominent increase in the scratch ratio (Fig. 3a), but a remarkable decrease in the numbers of invasive cells and numbers of tubes (Fig. 3b and c). Together, downregulation of GTSE1 suppressed migration, invasion and angiogenesis of NPC cells.

### GTSE1 upregulated the expression of STMN1 via FOXM1

**Fig. 4:**
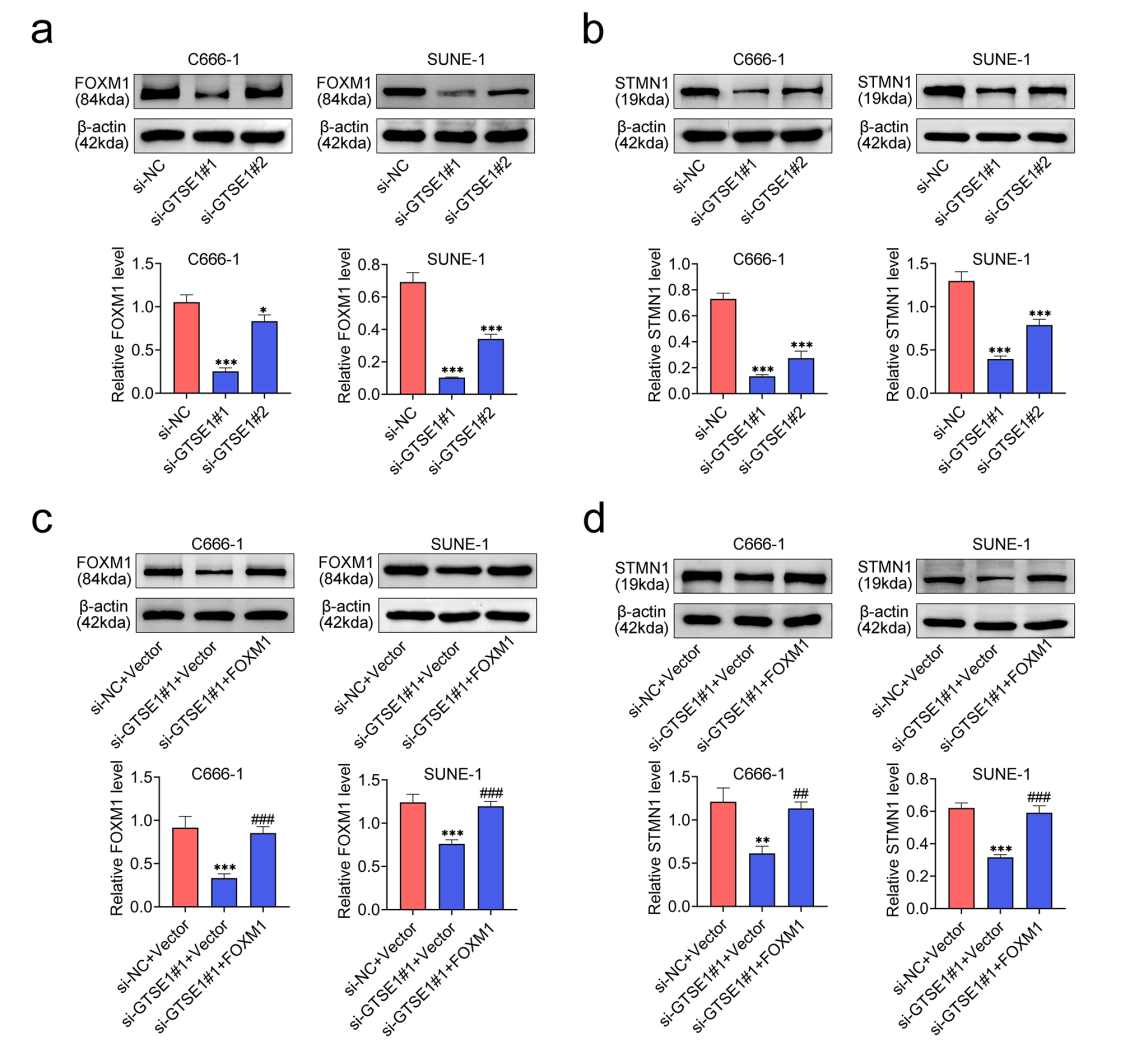
GTSE1 upregulated the expression of STMN1 by FOXM1. **(a)** The relative protein expression of FOXM1 was examined by western blot after C666-1 and SUNE-1 cells were transfected with si-GTSE1#1 and si-GTSE1#2. Data were normalized with β-actin. *p < 0.05 and ***p < 0.001 vs. si-NC. **(b)** The relative protein expression of STMN1 was detected by western blot after C666-1 and SUNE-1 cells were transfected with si-GTSE1#1 and si-GTSE1#2. Data were normalized with β-actin. ***p < 0.001 vs. si-NC. **(c)** The relative protein expression of FOXM1 was determined by western blot after C666-1 and SUNE-1 cells were transfected with si-GTSE1#1 and pcDNA vector plasmids containing FOXM1. Data were normalized with β-actin. ***p < 0.001 vs. si-NC + Vector; ###*p* < 0.001 vs. si-GTSE1#1 + Vector. **(d)** The relative protein expression of STMN1 was assessed by western blot after C666-1 and SUNE-1 cells were transfected with si-GTSE1#1 and pcDNA vector plasmids containing FOXM1. Data were normalized with β-actin. **p < 0.01 and ***p < 0.001 vs. si-NC + Vector; ##*p* < 0.01 and ###*p* < 0.001 vs. si-GTSE1#1 + Vector

Mechanically, FoxM1 is a transcriptional factor that plays important roles in the progression and development of various cancers, including NPC [30]. Thus, the level of FOXM1 was detected in NPC by western blot. The relative protein level of FOXM1 was notably decreased in both cells transfected with si-GTSE1#1 and si-GTSE1#2 (Fig. 4a). Moreover, STMN1 has been demonstrated to be essential for FoxM1-mediated proliferation of cancer cells, such as hepatocellular carcinoma cells, gastric cancer cells and colorectal cancer cells [31]. Here, we also found that transfection of both two siRNAs targeting GTSE1 into NPC cells evoked a conspicuous reduction in the relative protein level of STMN1 (Fig. 4b). To further investigate the role of FOXM1/STMN1 axis in si-GTSE1-mediated NPC cells, FOXM1 was overexpressed in both cells combined with the transfection of si-GTSE1#1. When si-GTSE1#1 was transfected into C666-1 and SUNE-1 cells, the relative protein expression of FOXM1 and STMN1 was notably diminished. However, FOXM1 overexpression significantly enhanced this expression (Fig. 4c and d). Altogether, GTSE1 upregulated the expression of STMN1 through FOXM1.

### GTSE1 promoted the malignant progression of NPC by upregulating STMN1

**Fig. 5:**
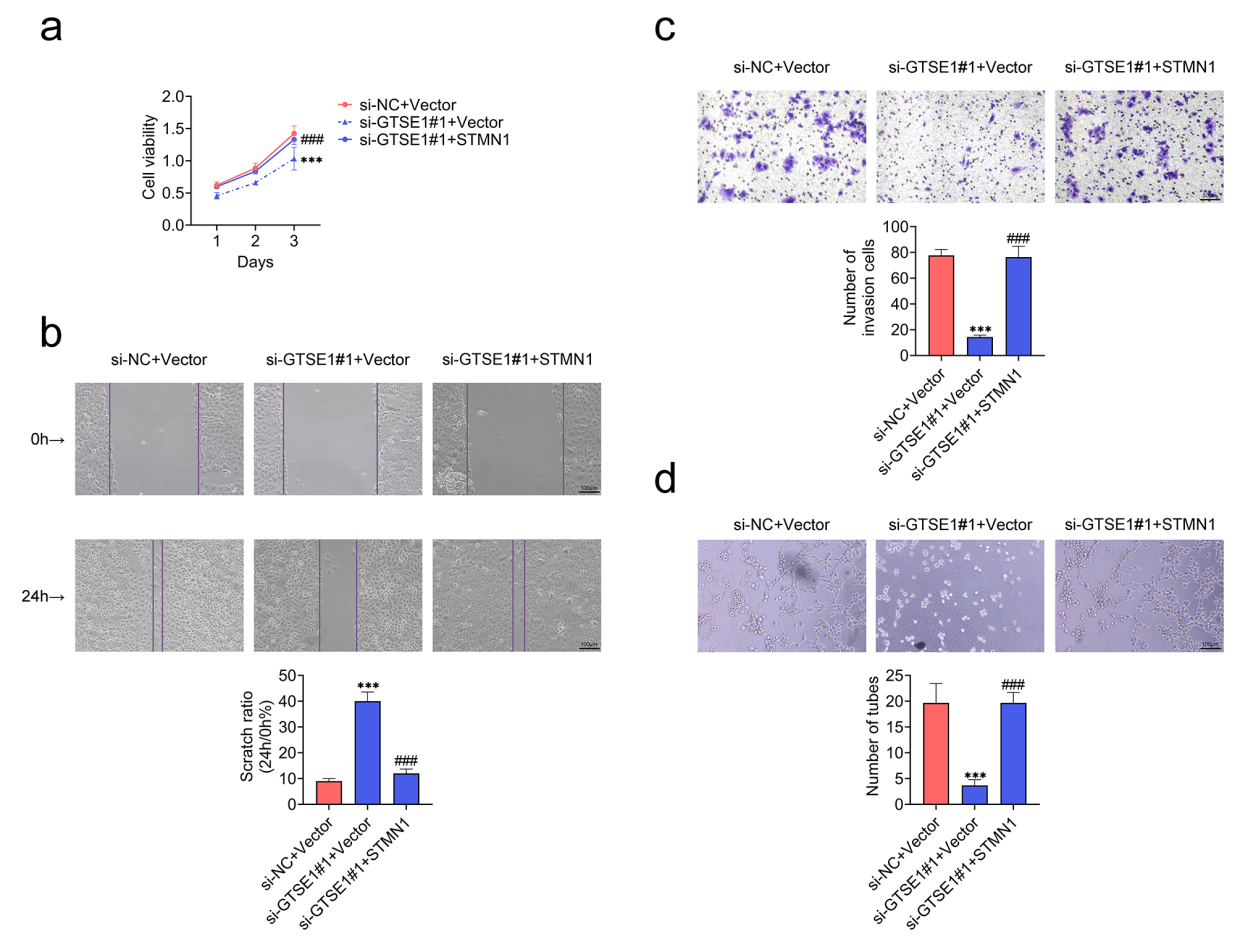
GTSE1 promoted the malignant progression of NPC by upregulating STMN1. C666-1 cells were transfected with si-GTSE1#1 and pcDNA vector plasmids containing STMN1. **(a)** Examination of cell viability by CCK-8. **(b)** The migration ability was assessed by the cell scratch wound-healing experiment. Scale bar = 100 μm. **(c)** The invasion ability was evaluated by transwell assays. Scale bar = 50 μm. **(d)** The angiogenesis ability was determined by the tube forming experiment. Scale bar = 100 μm. ***p < 0.001 vs. si-NC + Vector; ###*p* < 0.001 vs. si-GTSE1#1 + Vector

In order to validate the function of STMN1 in GTSE1-mediated advancement of NPC, si-GTSE1#1-transfected C666-1 cells were used to overexpress STMN1. Transfection of si-GTSE1#1 into C666-1 cells significantly reduced cell viability and enhanced the scratch ratio, which was prominently reversed with the STMN1 overexpression (Fig. 5a and b). Besides, the decrease in the numbers of invasive cells and numbers of tubes in C666-1 cells transfected with si-GTSE1#1 was markedly restored with the overexpression of STMN1 (Fig. 5c and d). Totally, GTSE1 enhanced the malignant progression of NPC through upregulating STMN1.

### Knockdown of GTSE1 inhibited the growth of NPC cells in vivo

**Fig. 6:**
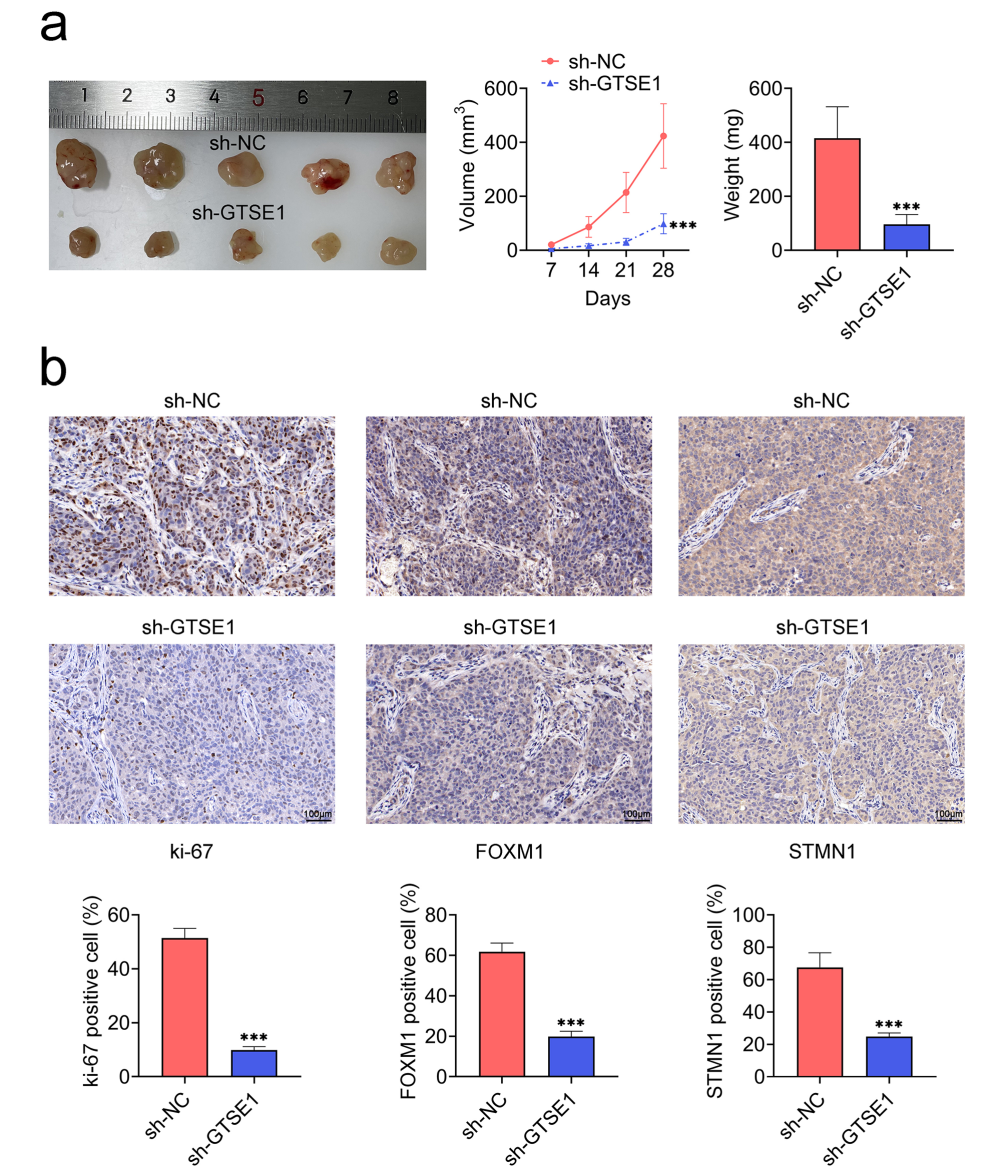
Knockdown of GTSE1 inhibited the growth of NPC cells in vivo. BALB/c nude mice were subcutaneously inoculated into the right flank with a total of 5 × 10^6^ of C666-1 cells transfected with sh-GTSE1 or sh-NC. **(a)** Tumor volume was monitored every seven days for consecutive four weeks and quantified by the formula: volume = 1/2×length×width^2^. After four weeks, the tumors samples were excised and weighed. **(b)** The expression levels of Ki-67, FOXM1 and STMN1 were examined by immunohistochemistry. Scale bar = 100 μm. ***p < 0.001 vs. sh-NC

Furthermore, the function of GTSE1 was evaluated in vivo by the subcutaneous inoculation of C666-1 cells transfected with sh-GTSE1 into nude mice. When compared to the mice with sh-NC, the tumor weight and volume were much lower in the GTSE1 knockdown mice (Fig. 6a). In addition, the expression level of Ki-67, FOXM1 and STMN1 in the GTSE1 knockdown mice was reduced compared with sh-NC mice (Fig. 6b). Thus, GTSE1 knockdown repressed the growth of NPC cells in vivo.

## Discussion

Nasopharyngeal carcinoma has a poor prognosis in southern China and Southeast Asia. To further improve the diagnosis and treatment of NPC, it is crucial to discover novel therapeutic targets. The function and mechanism of GTSE1 in NPC were investigated in the current study. The expression of GTSE1 was elevated in NPC tissues and cells. NPC cells invasion, migration, proliferation, and angiogenesis were all inhibited by GTSE1 knockdown. Mechanically, FOXM1 and STMN1 expression was downregulated by GTSE1 knockdown, but this was restored by FOXM1 overexpression. The function of GTSE1 silencing on the malignant development of NPC cells was reversed by overexpression of STMN1. Furthermore, the tumor volume and weight of xenografted mice were reduced by GTSE1 knockdown. Together, downregulation of GTSE1 repressed proliferation, migration, invasion and angiogenesis of NPC by upregulating STMN1.

A recent pan-cancer analysis revealed that GTSE1 is upregulated in different tumors, such as bladder cancer, breast cancer, colon adenocarcinoma, cholangiocarcinoma, cervical squamous cell carcinoma, glioblastoma multiforme, esophageal carcinoma, kidney renal papillary cell carcinoma, kidney chromophobe, kidney renal clear cell carcinoma, lung squamous cell carcinoma, lung adenocarcinoma, liver hepatocellular carcinoma, rectum adenocarcinoma, prostate adenocarcinoma, stomach adenocarcinoma, Sarcoma, uterine corpus endometrial carcinoma, thyroid carcinoma, as well as HNSC based on the data from TCGA [13]. Similar to this finding, our results demonstrated that the GTSE1 level was increased in NPC tissues and cells. Upregulation in the level of GTSE1 in NPC suggested that GTSE1 might play a role in the progression and development of NPC. Knock-down results showed that downregulation of GTSE1 suppressed proliferation, mobility, invasion and angiogenesis of NPC cells, and also inhibited the tumor growth in the NPC xenografted mice. Similar results was observed that silencing of GTSE1 represses cell proliferation, mobility and invasion have been reported in non-small-cell lung cancer cells [21], clear cell renal cell carcinoma [19, 20], esophageal squamous cell carcinoma [27], colon cancer [32], bladder cancer [14], acral melanoma [15], and hepatocellular carcinoma [17]. Collectively, knockdown of GTSE1 attenuated growth, mobility, invasion and angiogenesis of NPC cells.

Several studies have revealed that GTSE1 upregulates the expression level of FOXM1. Lai et al. [33] report that GTSE1 positively regulated the transcriptional level of FOXM1, downstream factors of FOXM1 (CCNB1 [34] and CCND1 [35]), and transcription factors of FOXM1 (HIF-1α [36], SP1 [37], and E2F1 [38]), and shFOXM1 or the FOXM1 inhibitor reverses the pro-proliferation effect of GTSE1 in prostate cancer. Liu and colleagues [14] reveal that knockdown of GTSE1 reduces the expression of FOXM1 and CCNB1 in bladder cancer, indicating that GTSE1 positively modulates the expression and level of FOXM1. Here, silencing of GTSE1 decreased the expression of FOXM1, in line with the above-mentioned studies. FOXM1 that is upregulated in NPC has been identified as potential therapeutic and prognostic marker of NPC [39, 40], which participates in the malignant progress of NPC, including cell cycle progression, proliferation, migration, invasion, apoptosis, angiogenesis, stemness, glycolysis, metastasis, and resistance [30, 41–50]. Moreover, FOXM1 directly binds to and positively regulates the expression of STMN1, which drives the tumorigenesis [31]. AKT/FOXM1/STMN1 axis in lung cancer also contributes to resistance to tyrosine kinase inhibitors [51]. In the current study, the decreased expression level of STMN1 caused by the downregulation of GTSE1 in NPC cells was recovered with the FOXM1 overexpression, consistently suggesting that FOXM1 positively modulated the level of STMN1 in NPC cells. STMN1, a microtubule-binding protein, binds to α/β-Tubulin heterodimers, thereby promoting the dissociation of microtubules or assembly suppression of microtubules [52]. STMN1, upregulated in NPC, is an independent prognostic factor in NPC [53], which is associated with drug resistance of NPC [54, 55], and radioresistance [56]. Furthermore, STMN1 participates in the proliferation, apoptosis, migration, and angiogenesis of NPC [57, 58]. Here, downregulation of GTSE1 decreased the expression of STMN1, and overexpression of STMN1 reversed the effects of the GTSE1 silencing on growth, mobility, invasion and angiogenesis of NPC cells. Therefore, the results demonstrated that downregulation of GTSE1 inhibited proliferation, mobility, invasion and angiogenesis of NPC by the upregulation of STMN1.

In summary, silencing of GTSE1 suppressed cell growth, mobility, invasion and angiogenesis of NPC by upregulating STMN1. However, several limitations still should be resolved in the future. Since GTSE1 has been identified to be strongly related to the prognosis of various tumors, the role of GTSE1 in the NPC prognosis should be addressed in the following study by collecting the clinical data of NPC patients. Besides, the function of GTSE1 on the other important malignant progression of NPC, such as apoptosis, glycolysis, metastasis, can be investigated in the further study. Moreover, the direct interaction between FOXM1 and STMN1 can be verify in NPC cells to solidify the results. Additionally, more experiments can be conducted to verify our conclusion, such as clone formation experiments. Moreover, the specific regulatory mechanisms of GTSE1 on FOXM1/STMN1 will be explored in the following study. Furthermore, the specific regulatory mechanisms of STMN1 on angiogenesis in NPC need to be explored in the future. Briefly, the results provide the pre-clinical evidence for the discovery of the potential target for the treatment of NPC.

## Materials and methods

### Tissue specimen

20 pair of tumor samples and adjacent para-carcinoma samples were collected from NPC patients at Ningbo Medical Center Lihuili Hospital. All of the patients were identified by pathological examination as having only one form of cancer. The Board and Ethics Committee of Ningbo Medical Center Lihuili Hospital approved this experiment (approval number: 2022 − 440), and each participant provided the written informed consent.

### Cell culture

Human nasopharyngeal epithelial cell line NP69 was acquired from Sigma-Aldrich (SCC197, St. Louis, MO, USA) and grown in keratinocyte serum-free media (17,005,042, Gibco, Rockville, MD, USA) with 10% fetal bovine serum (FBS, 10,082,147, Gibco) and 1% penicillin-streptomycin (P/S) solution (PB180120, Procell) in an incubator with 5% carbon dioxide (CO_2_) at 37 °C. NPC cell lines from American Type Culture Collection (ATCC, Manassas, VA, USA), such as C666-1 (ACS-5006) and SUNE-1 (CRL-5971) were purchased and grown in RPMI-1640 (30-2001, ATCC) with 10% FBS and 1% P/S at 37 °C with 5% CO_2_.

### Cell transfection

GenePharma (Shanghai, China) created the small interfering ribonucleic acid (siRNA) against GTSE1 (si-GTSE1) and the negative control (si-NC) to downregulate the level of GTSE1. The sequences of FOXM1 or STMN1 were introduced into pcDNA vector plasmids to upregulate the expression of FOXM1 or STMN1 based on the previous study [59]. C666-1 and SUNE-1 cells were transfected with si-GTSE1, si-NC, pcDNA vector plasmids containing FOXM1 (designated as FOXM1), pcDNA vector plasmids containing STMN1 (designated as STMN1), and the empty vector plasmids (designated as Vector) using Lipofectamine 3000 (L3000001, Invitrogen, Carlsbad, CA, USA). Cells were collected for ensuring studies 48 h after the transfection. \.

### Cell counting kit-8 (CCK-8) assay

A density of 5 × 10^3^ transfected cells were seeded into 96-well plates, where they were cultured at 37 °C with 5% CO_2_. Each well was added with 10 µl CCK-8 reagents (CA1210, Solarbio, Beijing, China) and incubated for 2 h at 37 °C to determine the cell viability as the previous description [60]. Using a microplate reader (Thermo Fisher Scientific, Waltham, MA, USA), the absorbance was measured at 450 nm.

### The 5-ethynyl-2′-deoxyuridine (EdU) incorporation experiment

6 × 10^5^ cells in each well were plated into 6-well plates, and the cells were cultured at 37 °C with 5% CO_2_. To assess the capacity for cell proliferation, the cells were stained using a BeyoClick™ EdU Cell Proliferation Kit with Alexa Fluor 647 (C0081S, Beyotime, Shanghai, China). Hoechst 33,342 (5 µg/mL, C0031, Solarbio) was used to identify cell nucleus. Fluorescence microscopy (Olympus, Tokyo, Japan) was used to capture the images.

### Cell scratch wound-healing experiment

Transfected cells were seeded into 6-well plates at a density of 6 × 10^5^ cells per well, and they were then cultivated at 37 °C until the confluence reached 95%. The scratch wound was created using a 200-µL pipette tip. The images were captured using an inverted microscope (Olympus) after 24 h. The scratch ratio was defined as the ratio of the scratch width at 24 h and the scratch width at 0 h.

### Transwell assays

Transwell assays were used to measure the invasion of NPC cells based on the prior reports [61, 62]. Transfected cells were resuspended into RPMI-1640 media at a density of 5 × 10^4^ cells/well without FBS, and Matrigel (356,234, Solarbio) was then added to the upper chamber of 24-well transwell plates (3422, Corning Company, New York, NY, USA). Media containing 10% FBS was distributed in the bottom chamber. A cotton swab was used to scratch the Matrigel after 24 h, and cells were subsequently fixed with 4% paraformaldehyde (P1110, Solarbio) and stained for 30 min with 0.1% crystal violet (G1062, Solarbio). An inverted microscope (Olympus) was used to capture images of the cells, and 10 randomly selected fields were used to tally the numbers of invaded cells.

### Tube forming experiment

Transfected NPC cells were cultured and the supernatant was isolated to incubate with human umbilical vein endothelial cells (HUVECs, C0035C, Gibco) with 5% CO_2_ at 37 °C. 50 µL supernatant containing Matrigel (M8371, Solarbio, diluted with supernatant at a ratio of 1:1) was plated into the 96-well plate, and then the 96-well plate was solidified in an incubator at 37 ℃. Subsequently, 100 µL HUVECs with a density of 3 × 10^5^ cells/mL were inoculated into the 96-well plate, and hatched at 37 ℃ for 4 h. ImageJ software (version 2.02, National Institutes of Health, USA) was used to assess the numbers of tubes after the images were captured using an Olympus microscope for photography.

### Animal experiment

Shanghai SLAC Laboratory Animal Co., LTD (Shanghai, China) supplied four-week-old BALB/c nude mice (4 weeks old). The mice were housed in a 12-hour light-dark cycle with a regulated temperature of 22–23˚C and fed under specified pathogen free (SPF) conditions. After two weeks, mice were randomly assigned into sh-NC group and sh-GTSE1 group with five mice in each group. A total of 5 × 10^6^ of C666-1 cells [63] transfected with the short hairpin RNA (shRNA) targeting to GTSE1 (sh-GTSE1) (GenePharma) were subcutaneously injected to mice right flank in the sh-GTSE1 group, whereas mice in sh-NC group received the same dose of C666-1cells transfected with the scrambled shRNA (sh-NC). For a span of four weeks, the tumor volume was measured every seven days using the following formula: volume = 1/2×length×width^2^. Mice were sacrificed after four weeks by inhaling excess isoflurane (R510-22, RWD, Shenzhen, China). Samples of the tumors were removed and weighed. The Animal Research Ethics Committee of Ningbo Medical Center Lihuili Hospital approved all animal experiments (approval number: 2022 − 440) and they were conducted in accordance with the Guide for the Care and Use of Laboratory Animals [64].

### Immunohistochemistry

Tumor tissues were fixed in 4% paraformaldehyde, and dehydrated with gradient ethanol. Then, tissues were embedded into paraffin (YA0011, Solarbio) and cut into sections with a thickness of 5 μm. The restoration was executed with sodium citrate buffer (pH 6.0, P0081, Beyotime) at 94 °C for 15 min. Subsequently, sections were sealed with 1% bovine serum albumin (BSA, ST2249, Beyotime) for one hour, and hatched with primary antibodies targeting Ki-67 (1:200, ab15580, Abcam), FOXM1 (1:250, ab207298, Abcam) and STMN1 (1:2000, ab52630, Abcam) overnight at 4 °C. The secondary antibody HRP labeled anti-rabbit IgG antibody (ab288151, Abcam) was used to incubate with sections at 37 °C for 30 min. The sections were re-stained with hematoxylin (G1080, Solarbio), and pictured under a light microscope (Olympus).

### Western blotting

Based on the previous studies [65], using RIPA lysis buffer (R0010, Solarbio), total proteins were isolated from NPC tissues and cells. BCA kit (PC0020, Solarbio) was then used to quantify the extracted proteins. 20 µg of protein samples were electrophoresed with sodium dodecyl sulfate-polyacrylamide gel electrophoresis (SDS-PAGE) and then transferred onto PVDF membranes (IPVH00010, EMD Millipore, Billerica, MA, USA). Following a one-hour room temperature blockade using blocking buffer (SW3015, Solarbio), primary antibodies such as anti-GTSE1 (1:1000, PA5-26879, Invitrogen), anti-FOXM1 (1:2000, ab180710, Abcam), anti-STMN1 (1:500, ab52630, Abcam) and anti-β-actin (1:1000, ab8227, Abcam) were applied to membranes for an overnight period at 4˚C. Subsequently, the membranes were developed using ECL solution (SW2030, Solarbio) after being incubated for an hour at room temperature with the Goat Anti-Rabbit IgG H&L (HRP) (1:20000, ab6721, Abcam). ImageJ software was used to measure the band intensity.

### Statistical analysis

The standard deviation (SD) was used to express the data as mean. The Student’s *t*-test or one-way analysis of variance (ANOVA) were used to determine whether there was statistically significant difference, followed by *post hoc* Bonferroni test using SPSS 26.0 software (IBM, Armonk, New York, USA). When *p* < 0.05, a significant difference was specified.

### Electronic supplementary material

Below is the link to the electronic supplementary material.

Supplementary Material 1

## Data Availability

All data generated or analyzed during this study are included in this published article. The datasets used and/or analyzed during the present study are available from the corresponding author on reasonable request.
