# Supplementary material for: GTSE1 promotes nasopharyngeal carcinoma proliferation and angiogenesis by upregulating STMN1
Source: Cell Div. 2024 May 2;19:16. doi: 10.1186/s13008-024-00119-9 (PMC11064356; doi:10.1186/s13008-024-00119-9)

Fig1b---[（1）Normal（2）Tumor]--- GTSE1


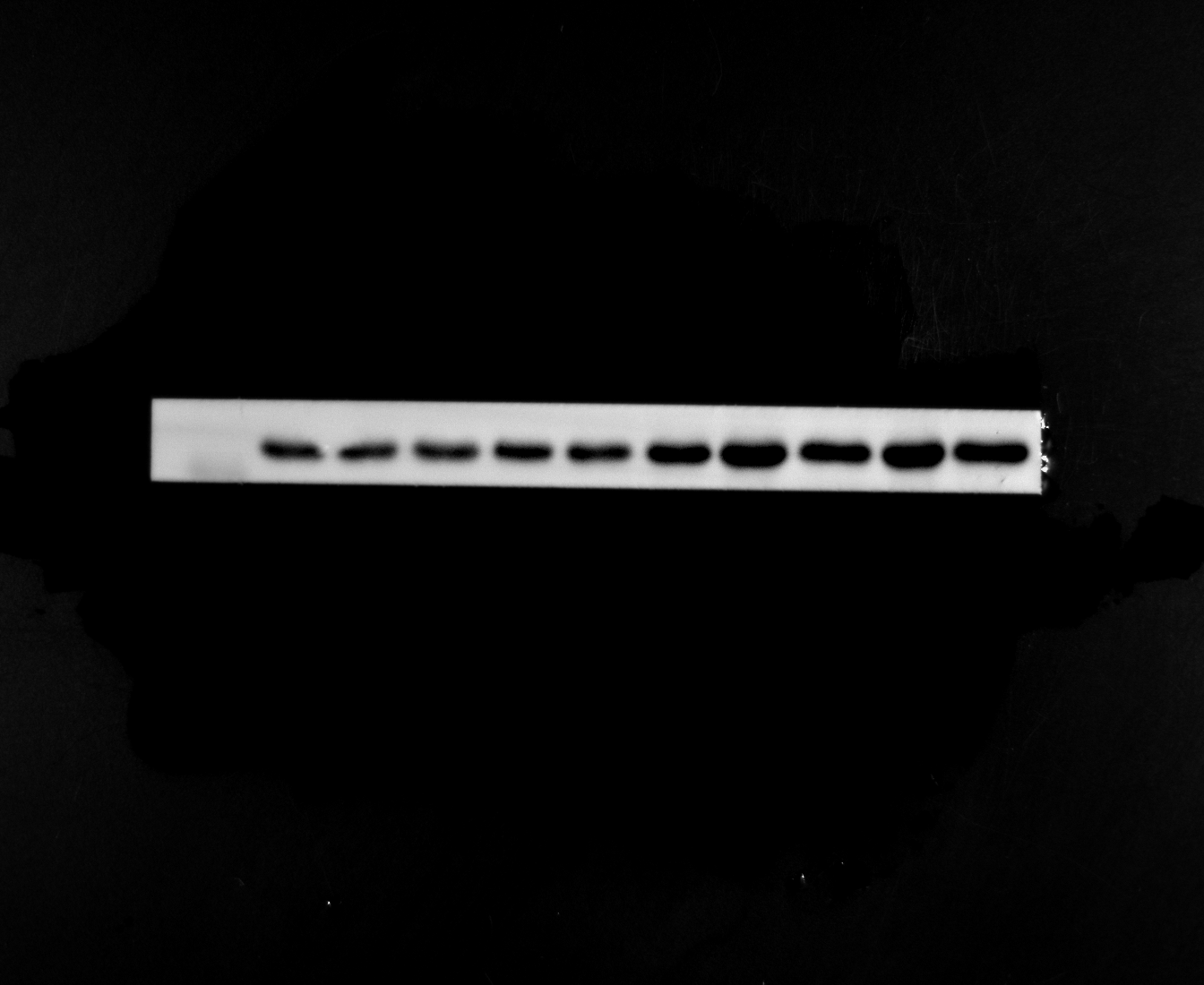


Fig1b---[（1）Normal（2）Tumor]--- β-actin


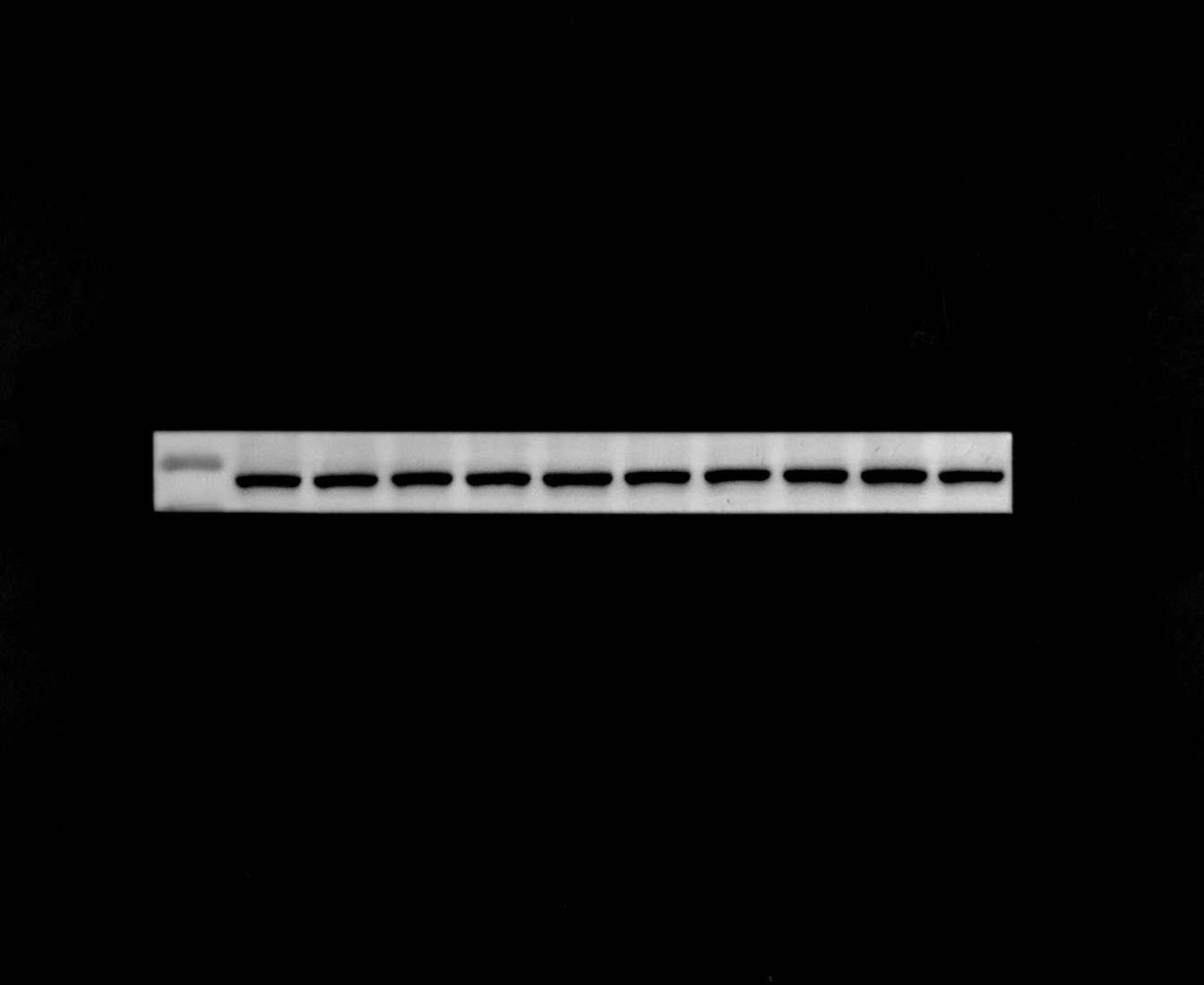


Fig1c---[（1）NP69（2）C666-1（3）SUNE-1]--- GTSE1


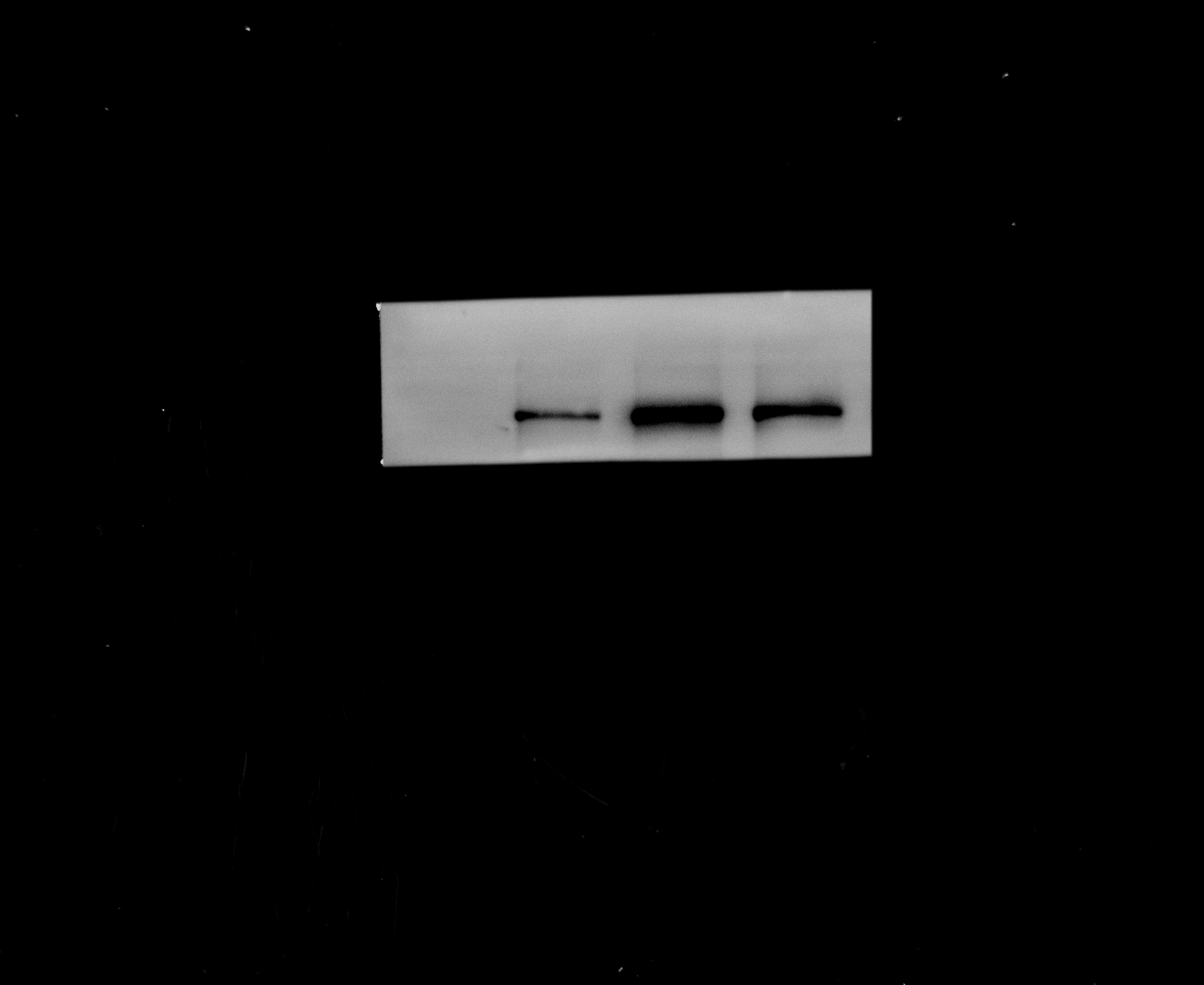


Fig1c---[（1）NP69（2）C666-1（3）SUNE-1]--- β-actin


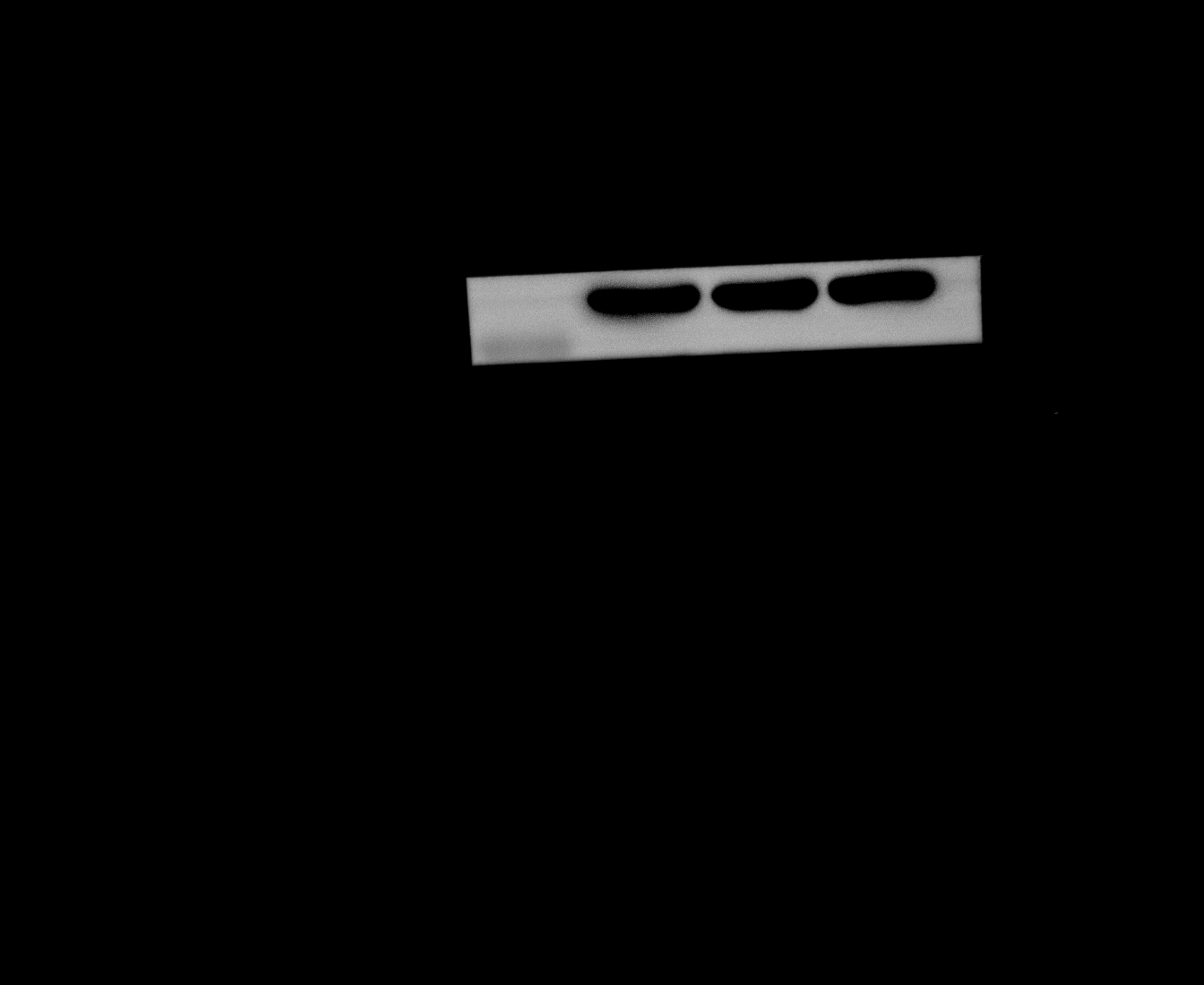


Fig2a C666-1---[（1）si-NC（2）si-GTSE1#1（3）si-GTSE#2]--- GTSE1


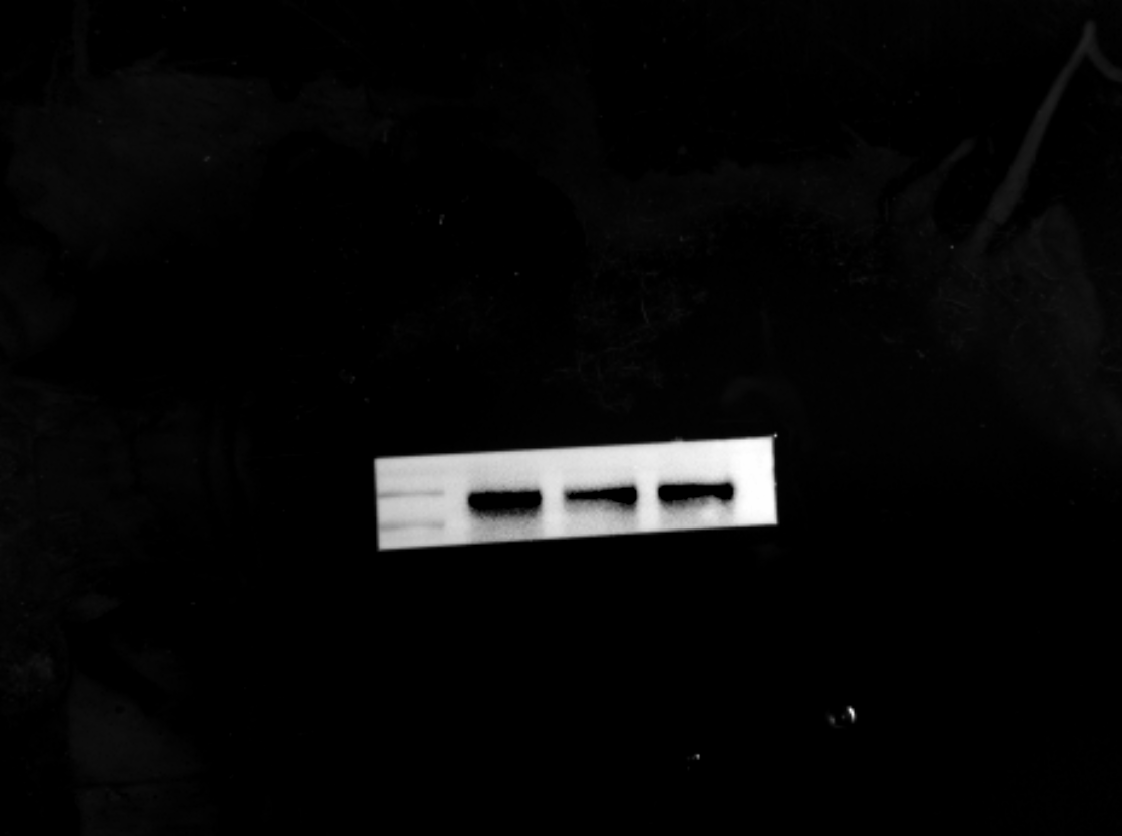


Fig2a C666-1---[（1）si-NC（2）si-GTSE1#1（3）si-GTSE#2]--- β-actin


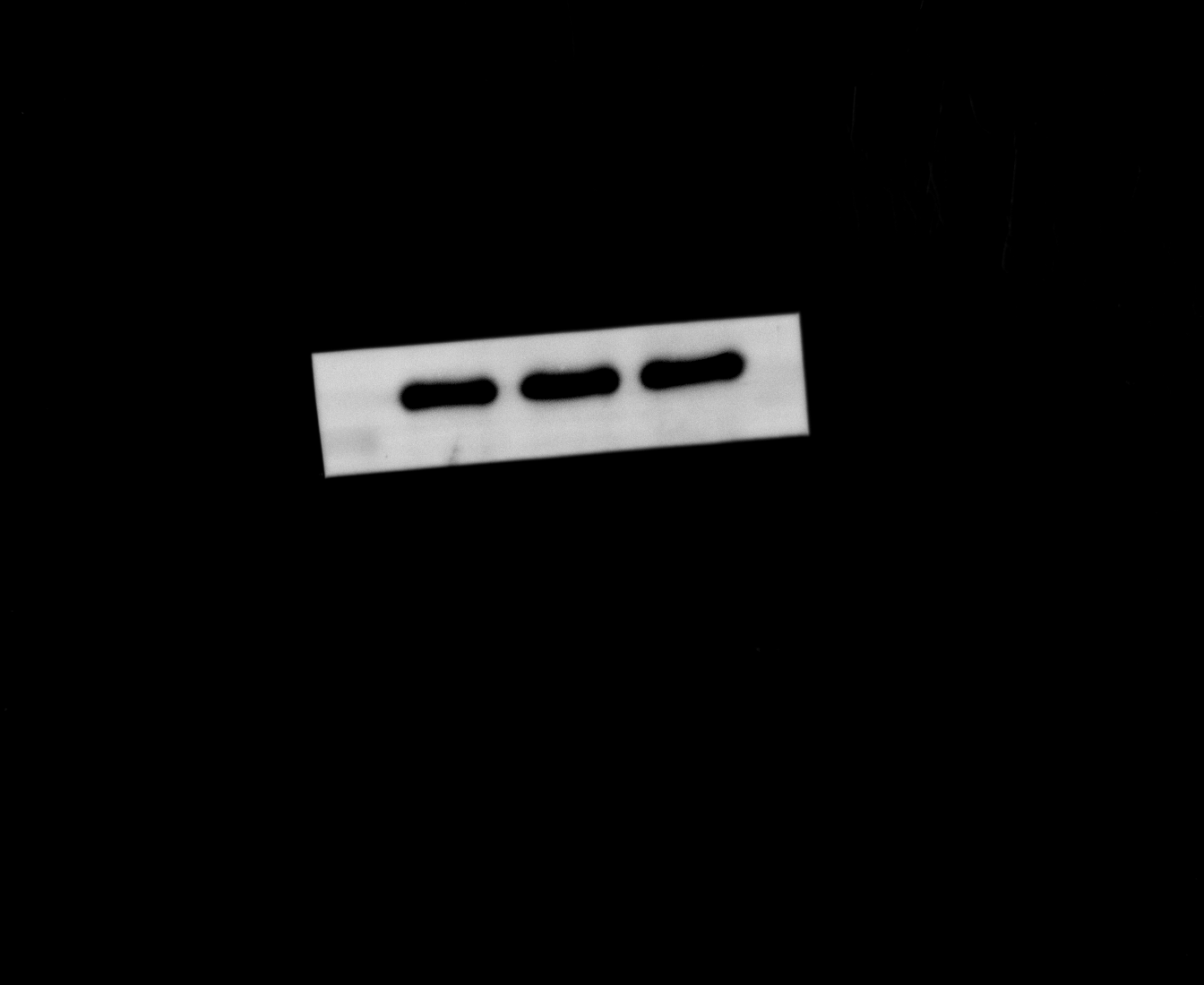


Fig2a SUNE-1---[（1）si-NC（2）si-GTSE1#1（3）si-GTSE#2]--- GTSE1


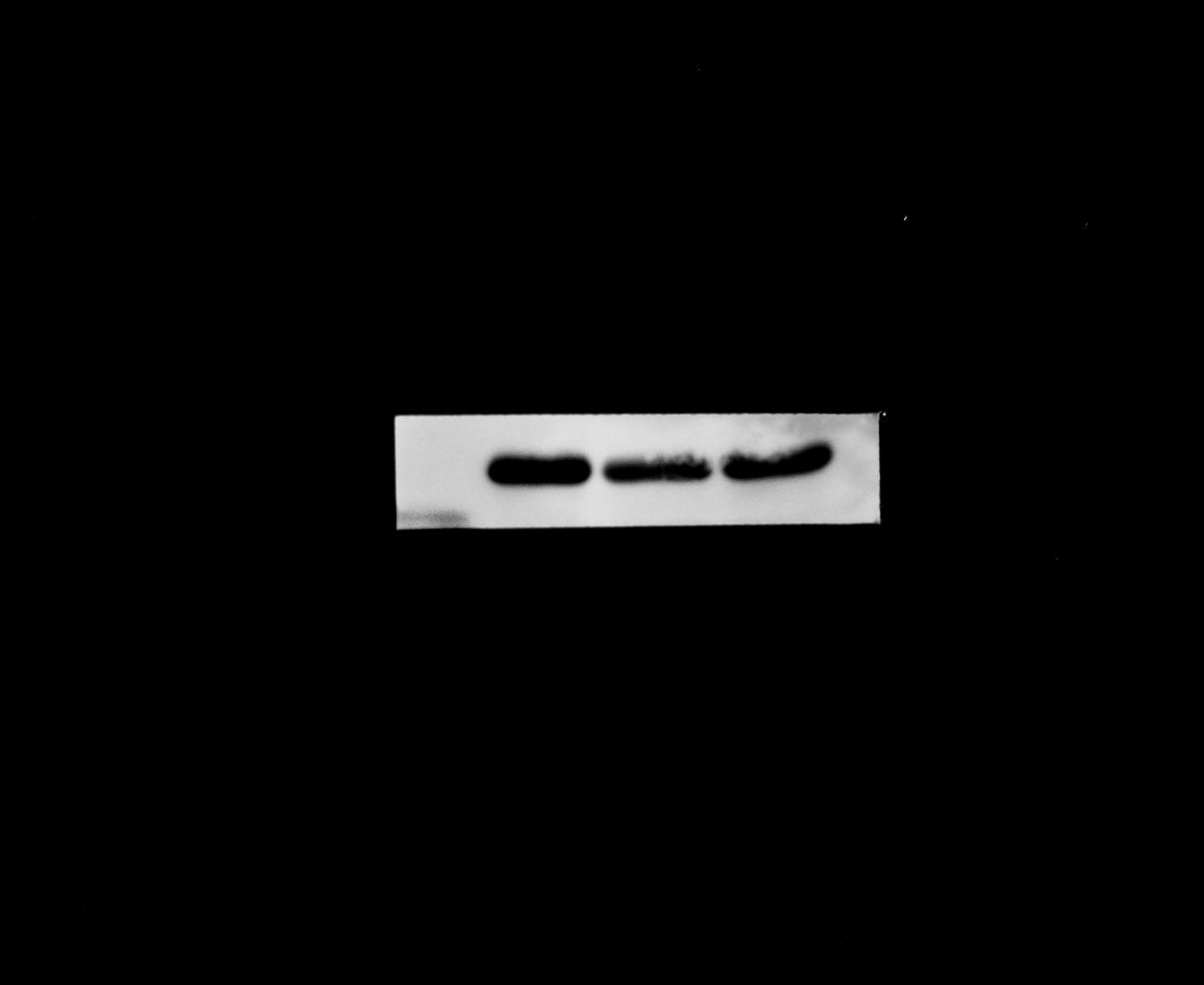


Fig2a SUNE-1---[（1）si-NC（2）si-GTSE1#1（3）si-GTSE#2]--- β-actin


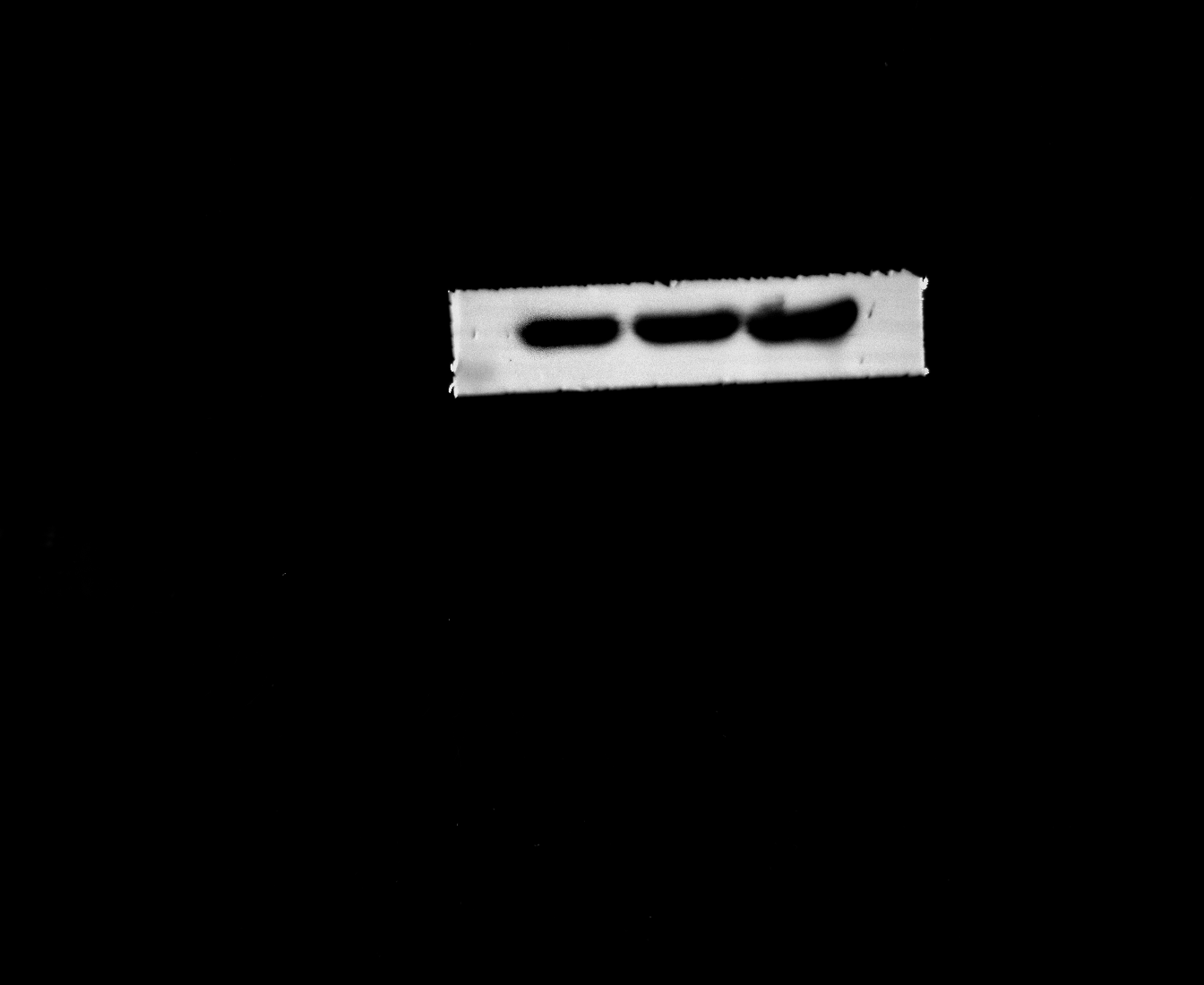


Fig4a C666-1---[（1）si-NC（2）si-GTSE1#1（3）si-GTSE#2]--- FOXM1


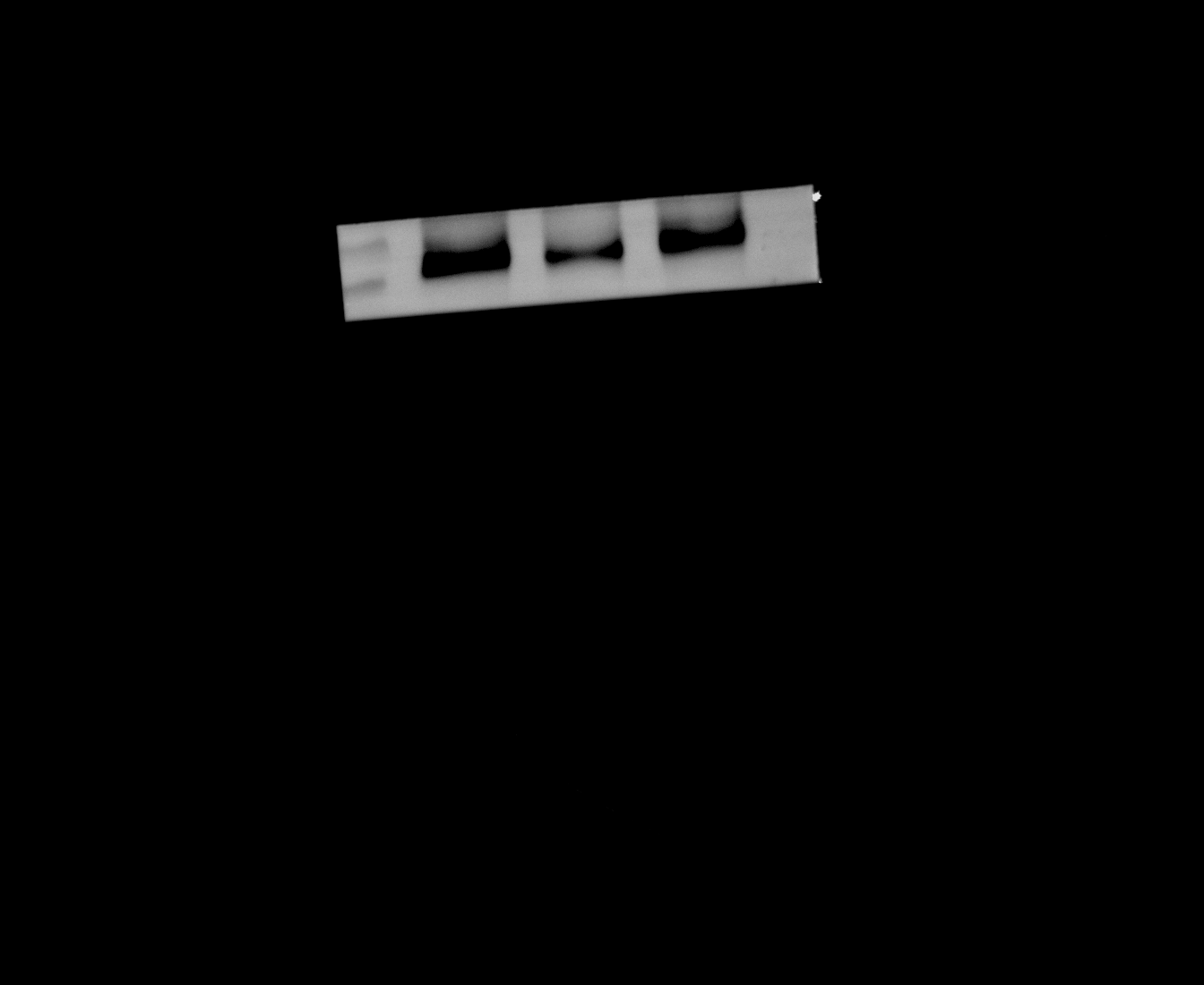


Fig4a C666-1---[（1）si-NC（2）si-GTSE1#1（3）si-GTSE#2]--- β-actin


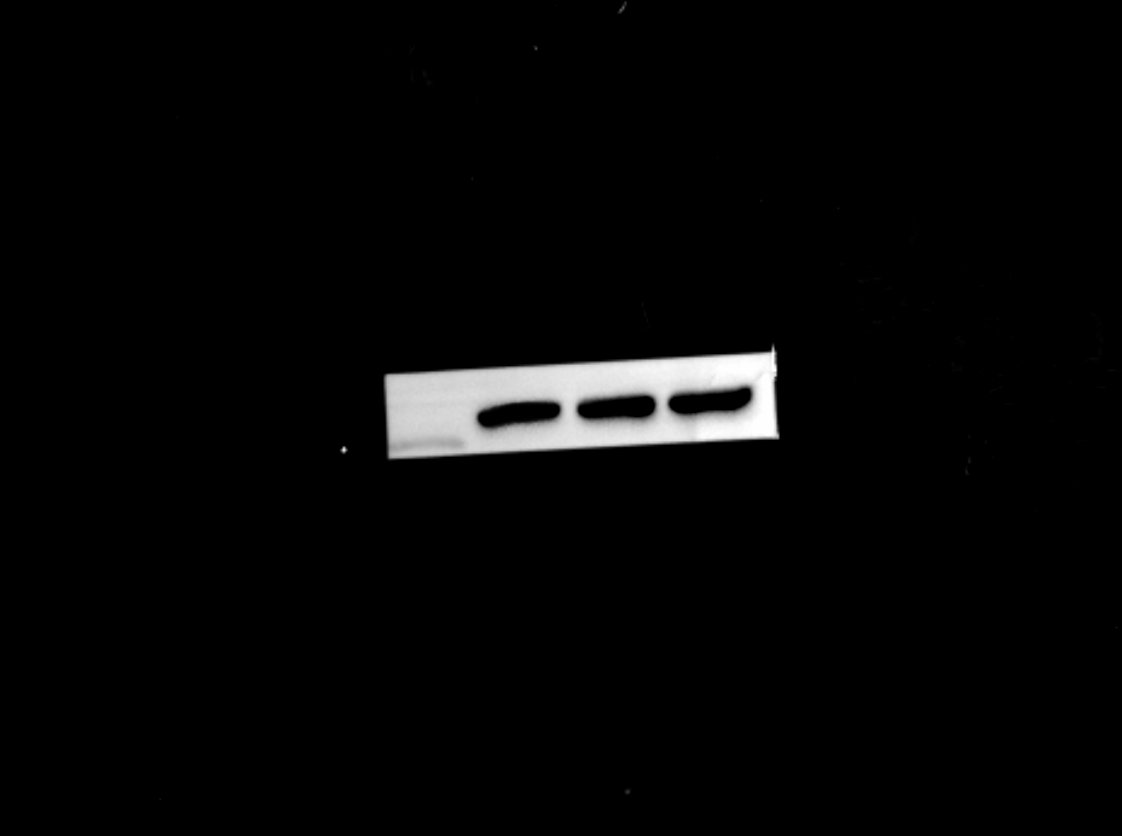


Fig4a SUNE-1---[（1）si-NC（2）si-GTSE1#1（3）si-GTSE#2]--- FOXM1


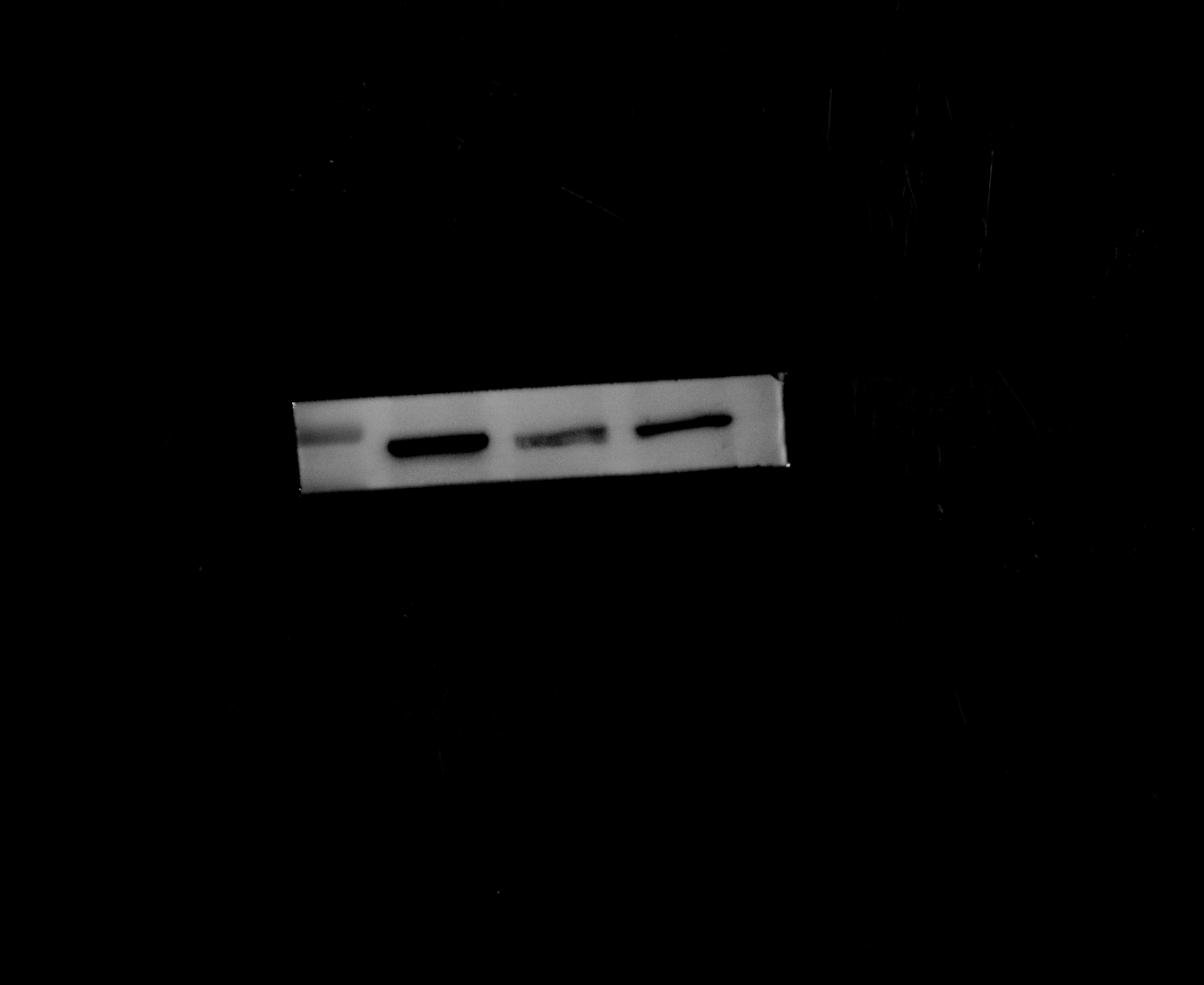


Fig4a SUNE-1---[（1）si-NC（2）si-GTSE1#1（3）si-GTSE#2]--- β-actin


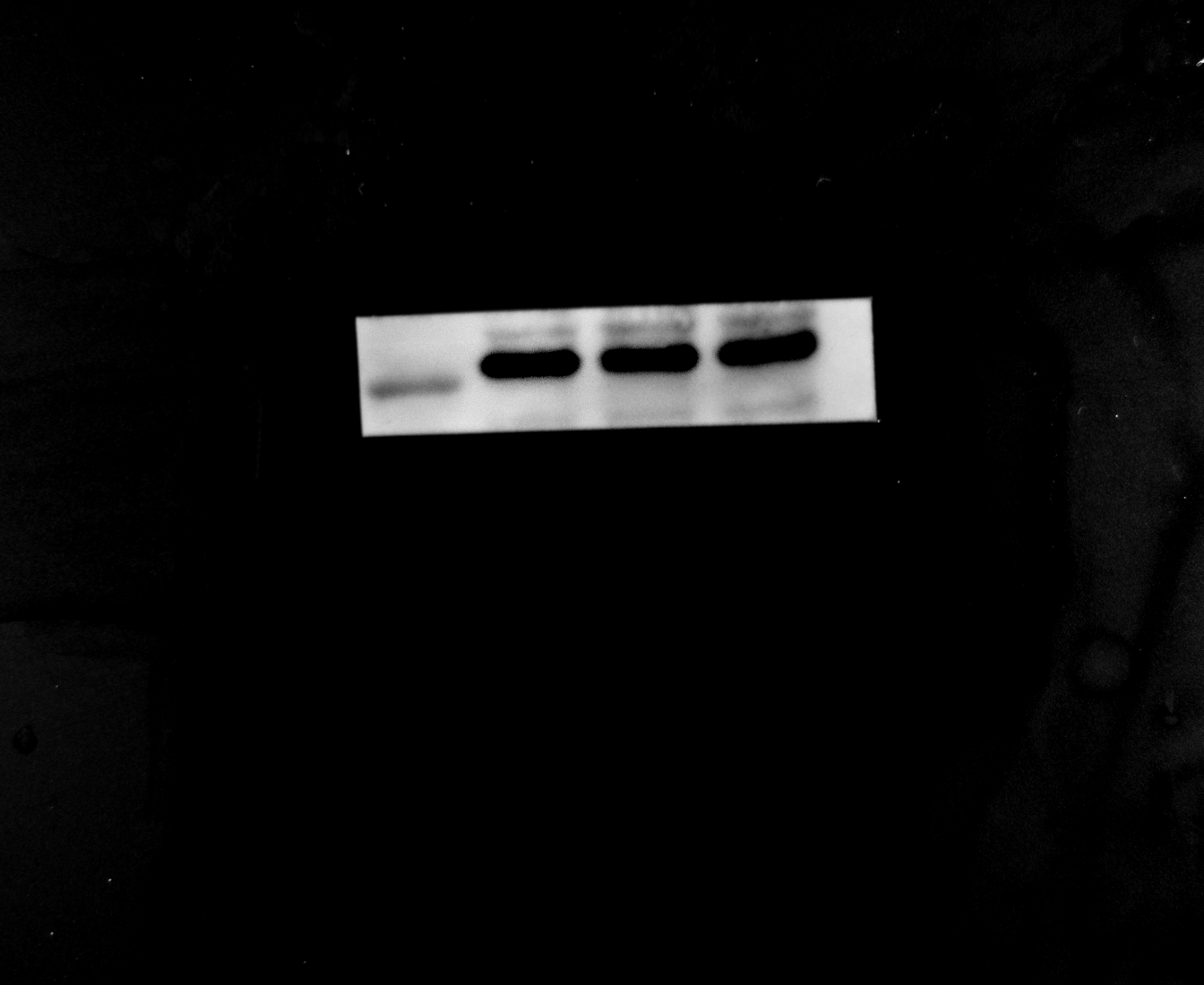


Fig4b C666-1---[（1）si-NC（2）si-GTSE1#1（3）si-GTSE#2]--- STMN1


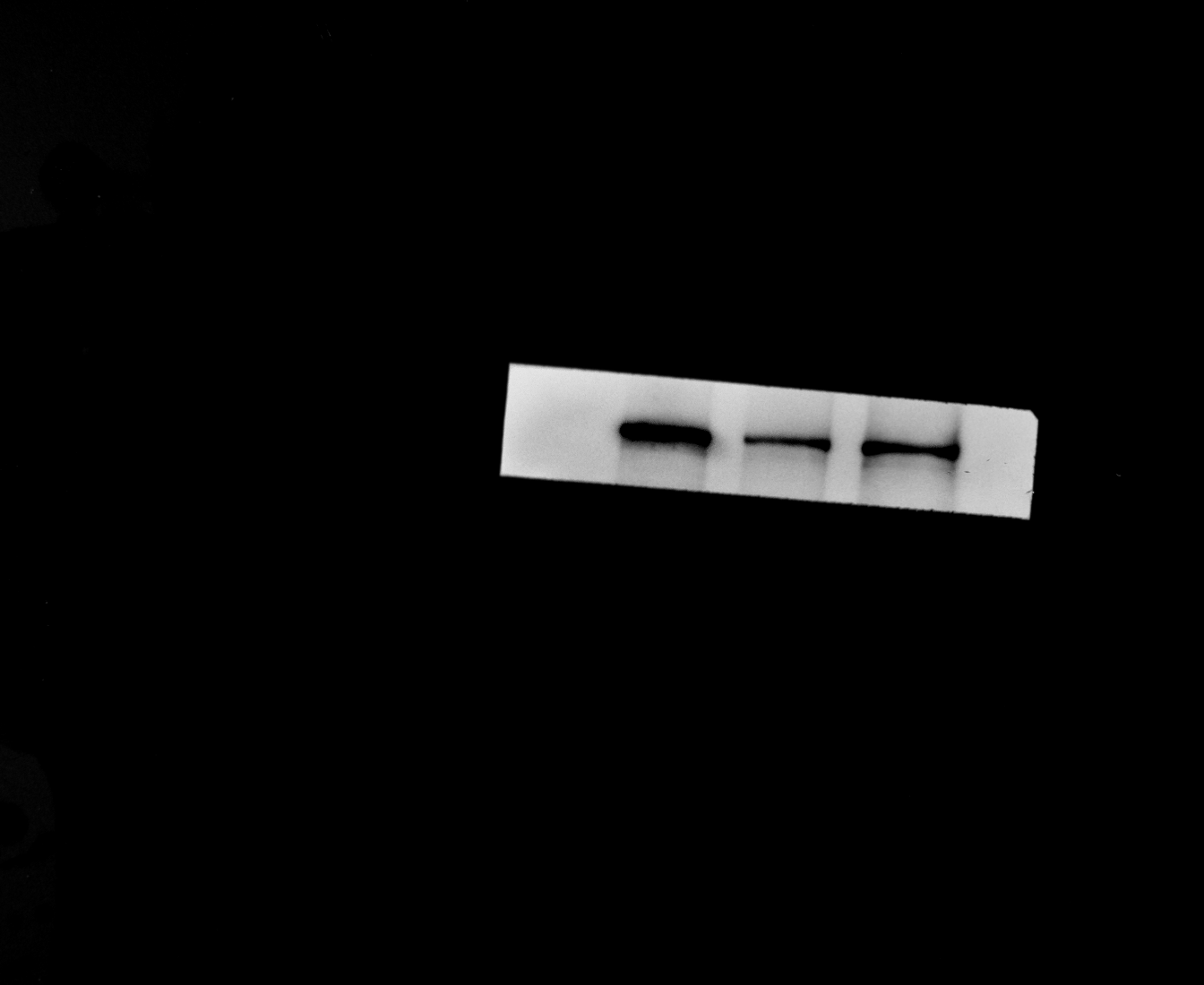


Fig4b C666-1---[（1）si-NC（2）si-GTSE1#1（3）si-GTSE#2]--- β-actin


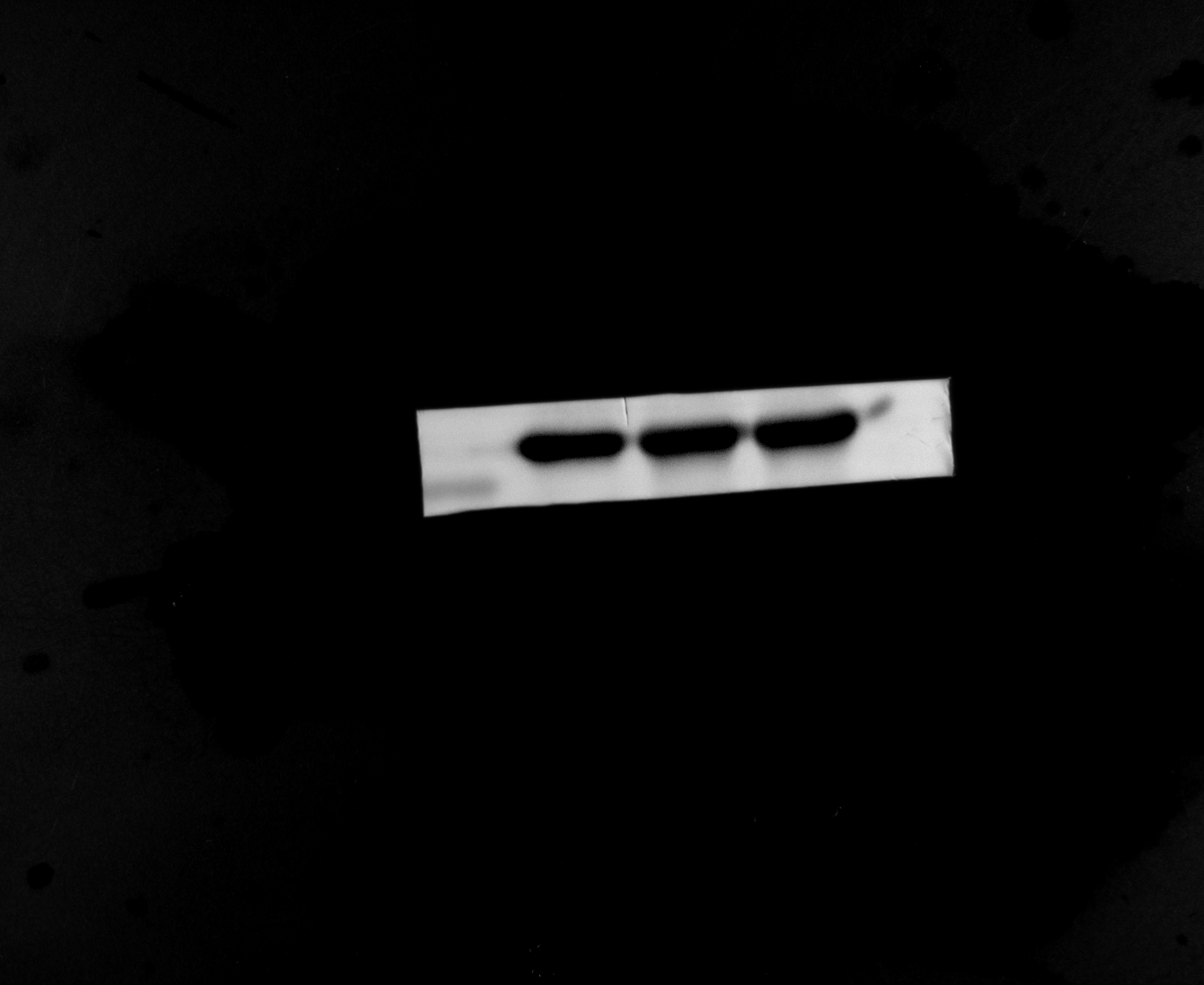


Fig4b SUNE-1---[（1）si-NC（2）si-GTSE1#1（3）si-GTSE#2]--- STMN1


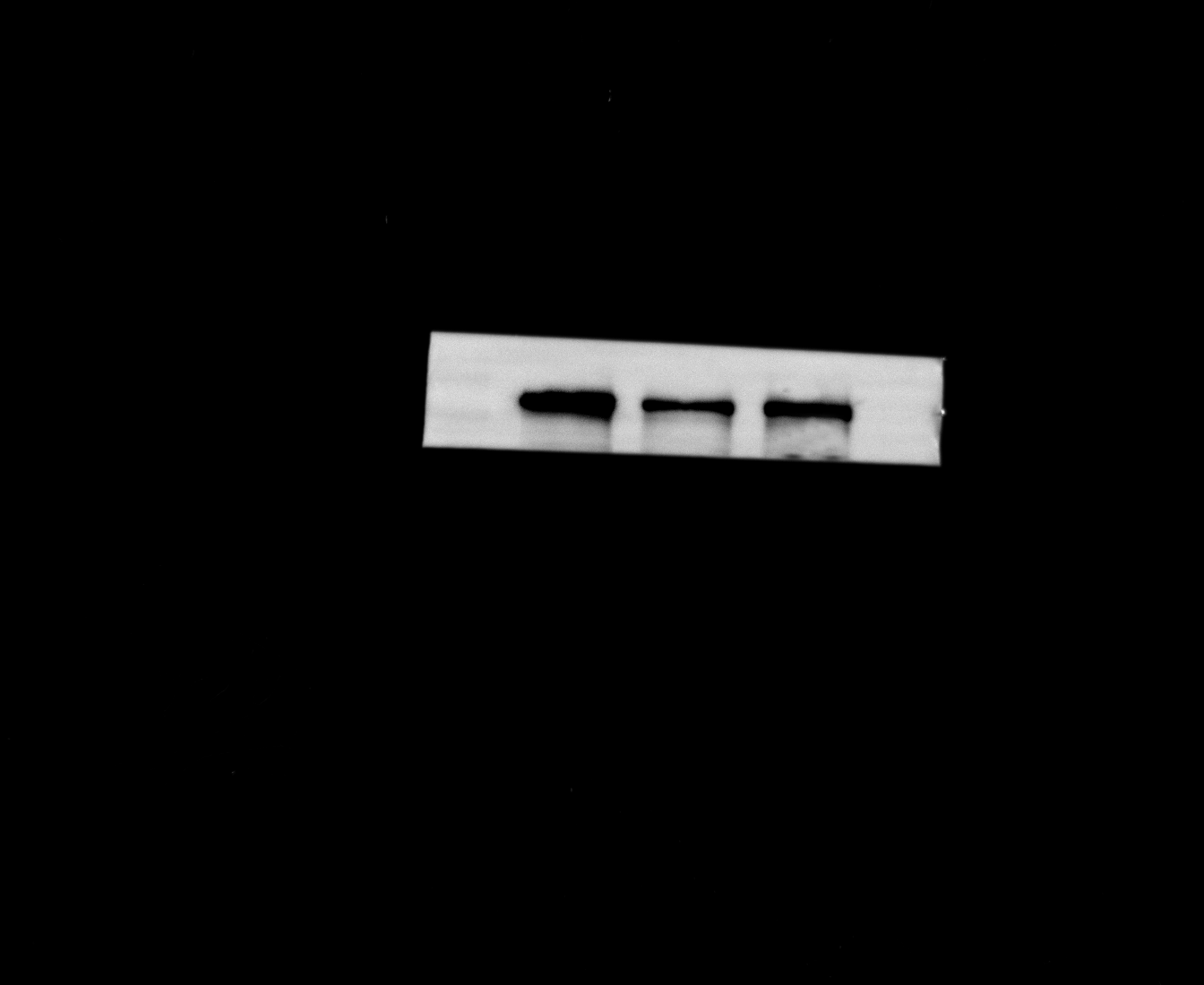


Fig4b SUNE-1---[（1）si-NC（2）si-GTSE1#1（3）si-GTSE#2]--- β-actin


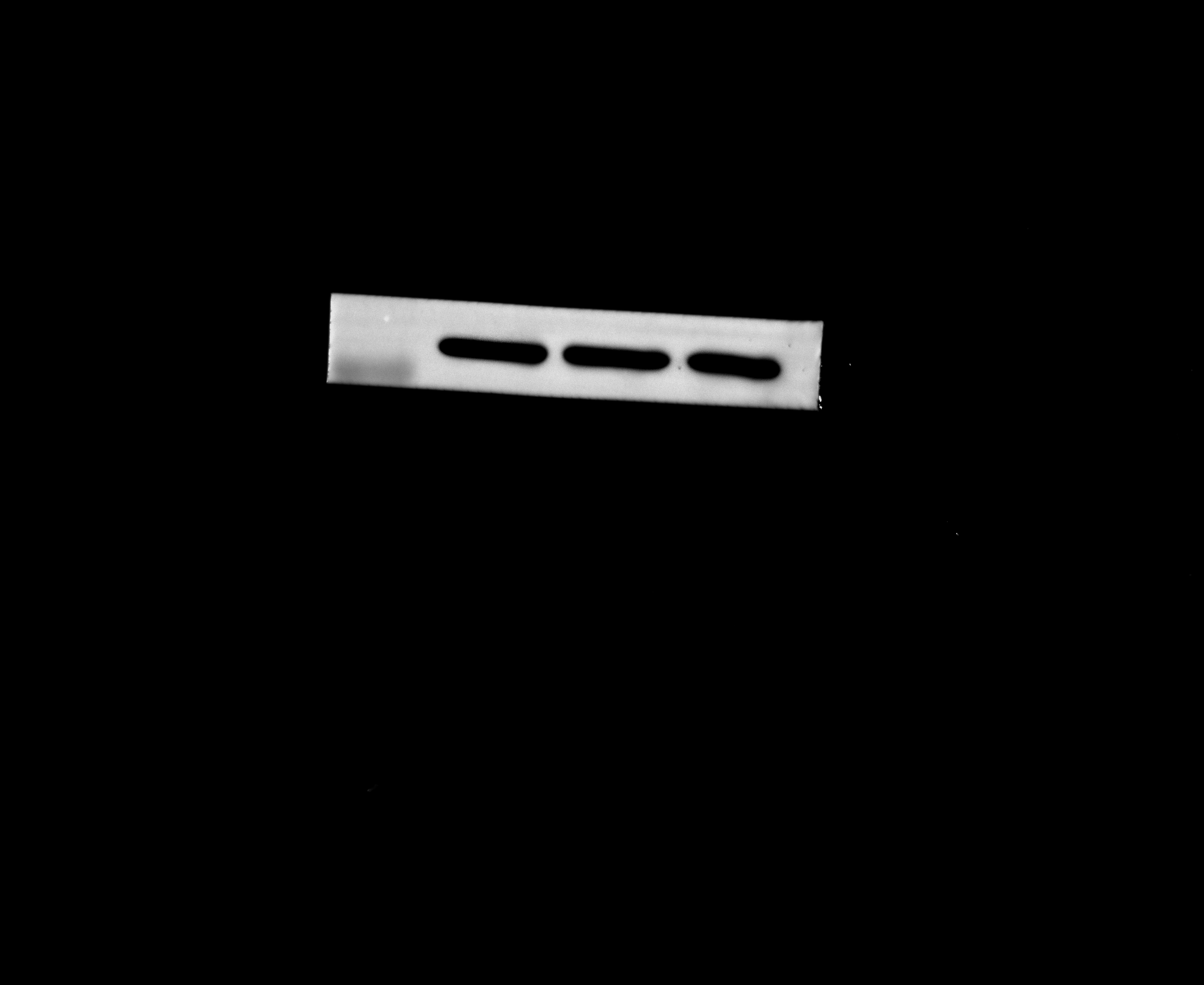


Fig4c C666-1---[（1）si-NC+vector（2）si-GTSE1#1+vector（3）si-GTSE#1+FOXM1]--- FOXM1


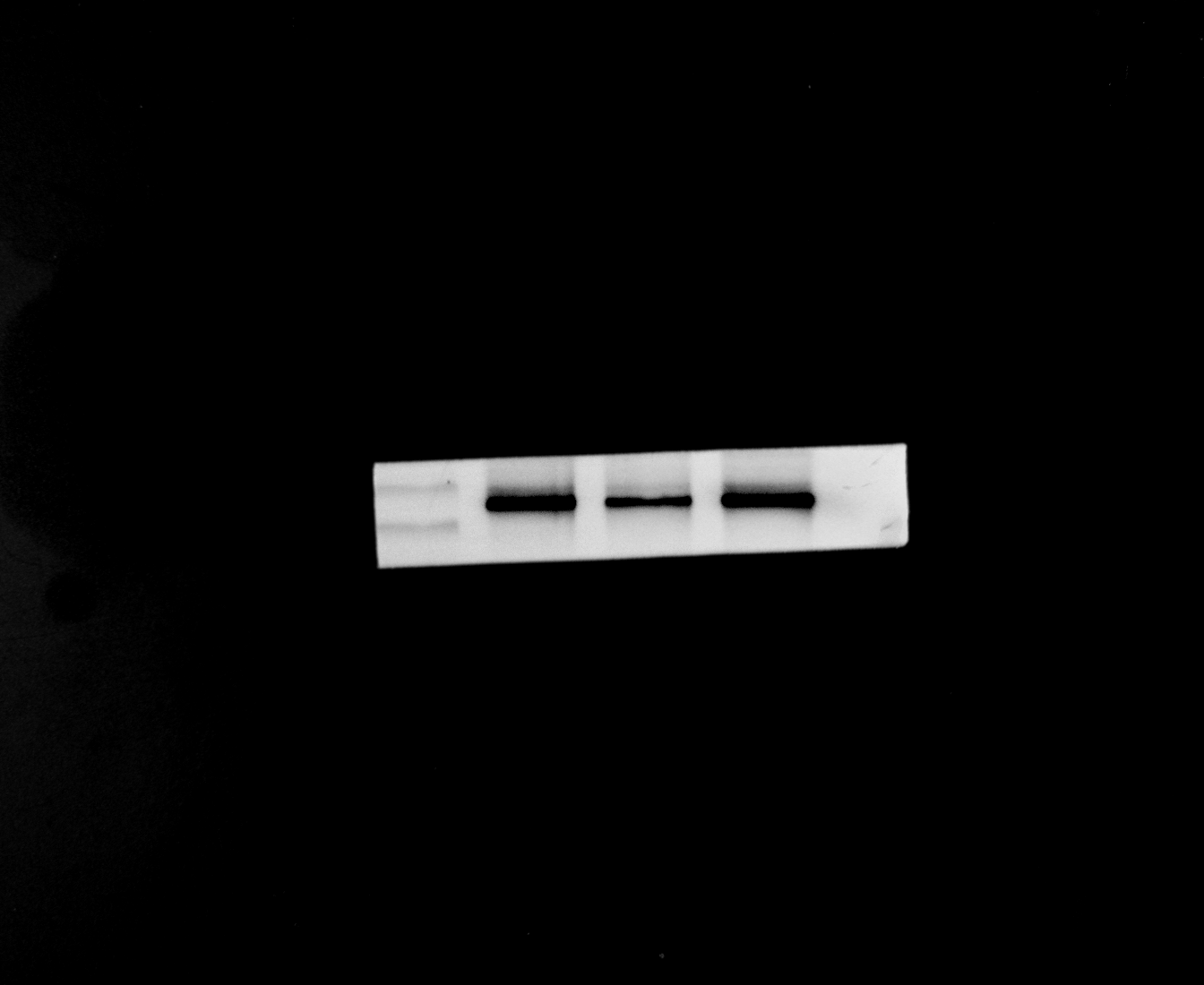


Fig4c C666-1---[（1）si-NC+vector（2）si-GTSE1#1+vector（3）si-GTSE#1+FOXM1]--- β-actin


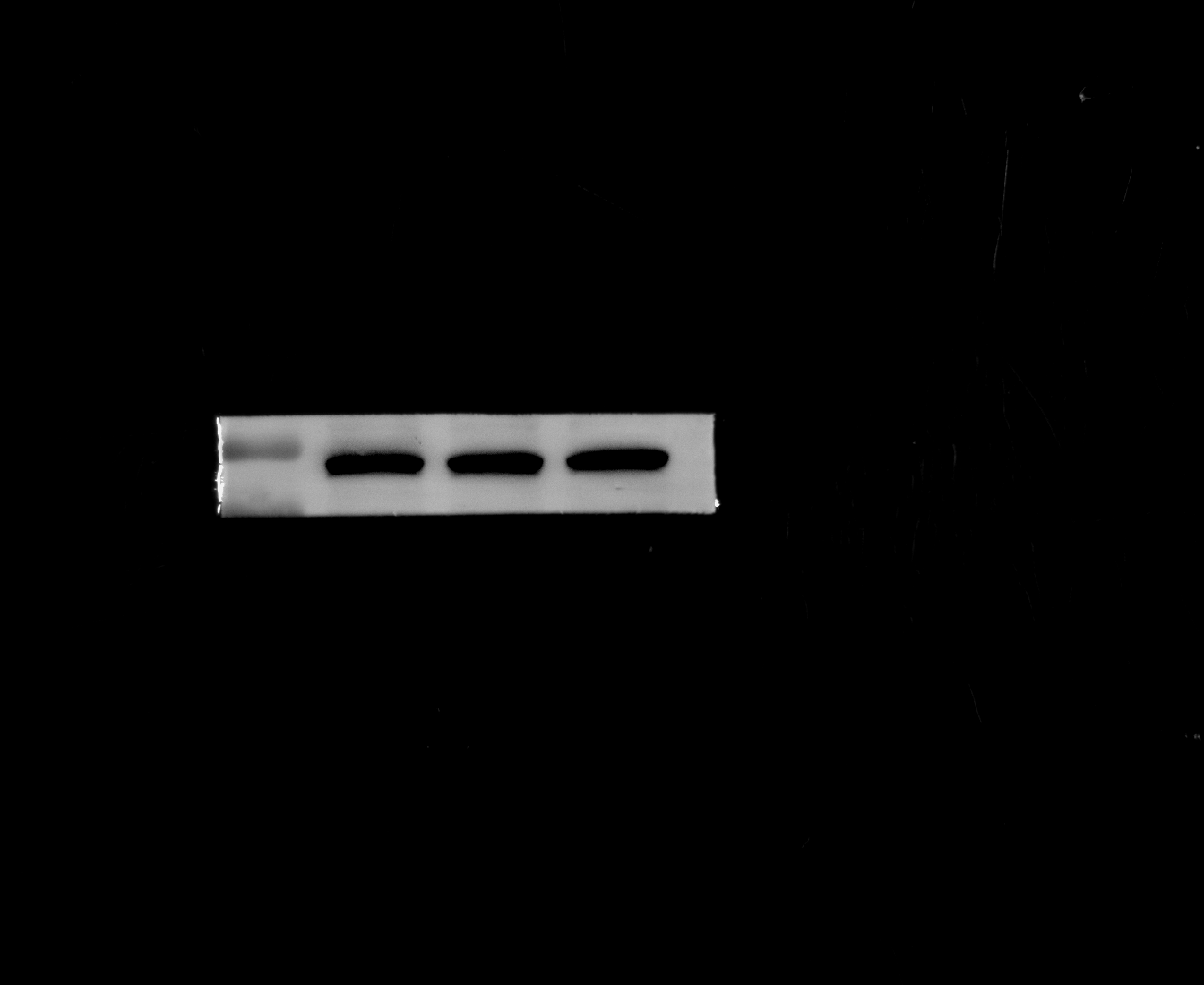


Fig4c SUNE-1---[（1）si-NC+vector（2）si-GTSE1#1+vector（3）si-GTSE#1+FOXM1]--- FOXM1


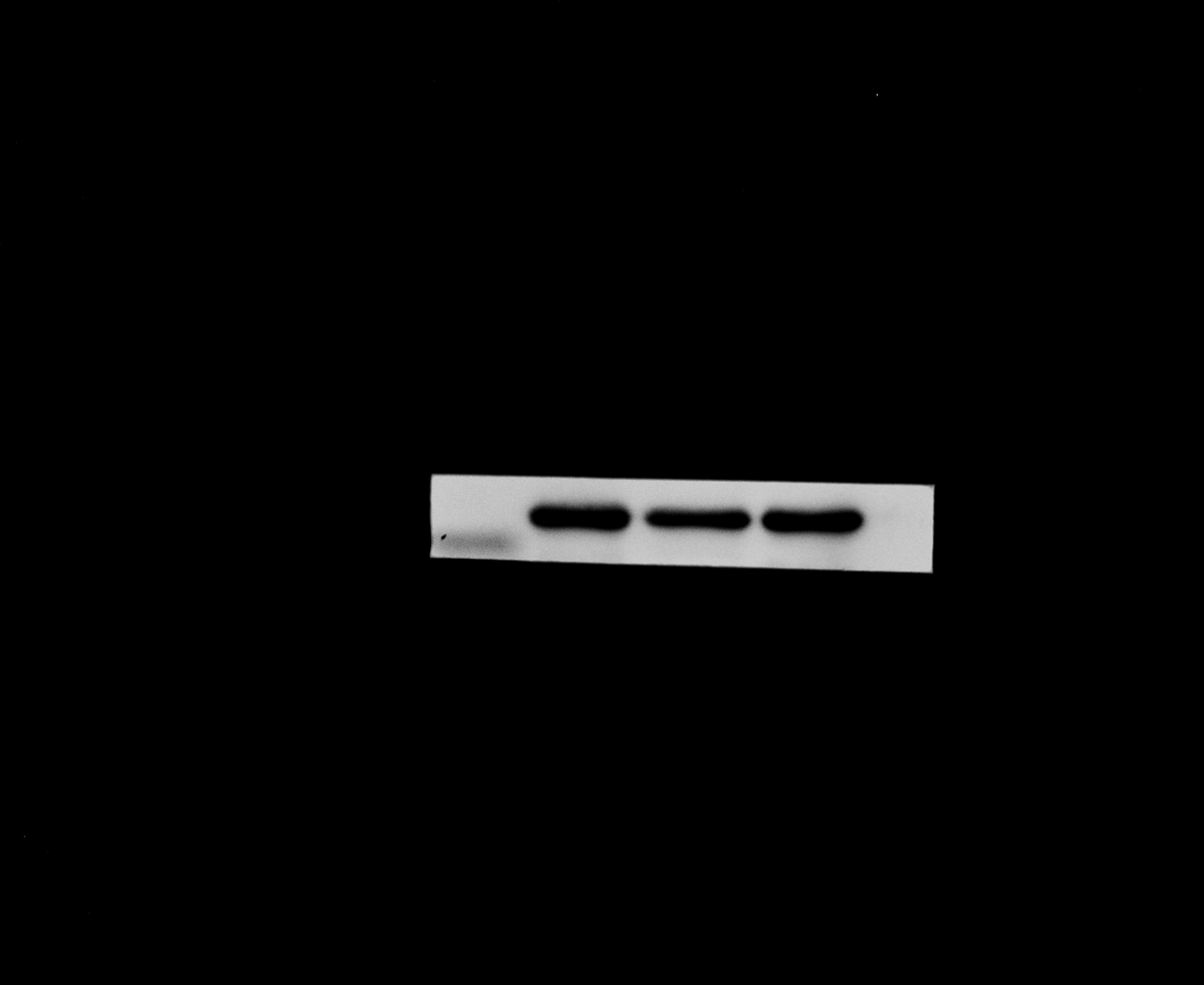


Fig4c SUNE-1---[（1）si-NC+vector（2）si-GTSE1#1+vector（3）si-GTSE#1+FOXM1]--- β-actin


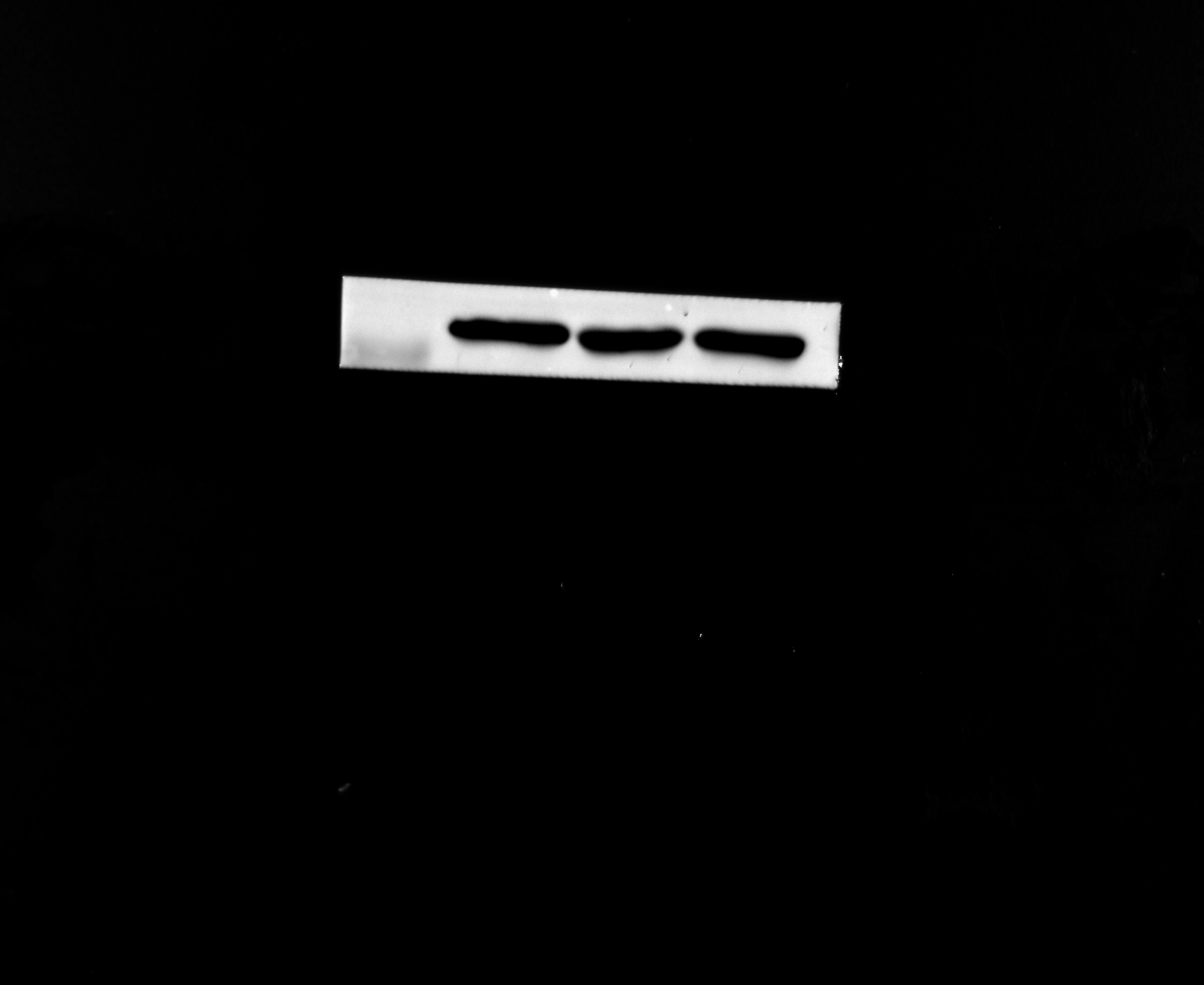


Fig4d C666-1---[（1）si-NC+vector（2）si-GTSE1#1+vector（3）si-GTSE#1+FOXM1]--- STMN1


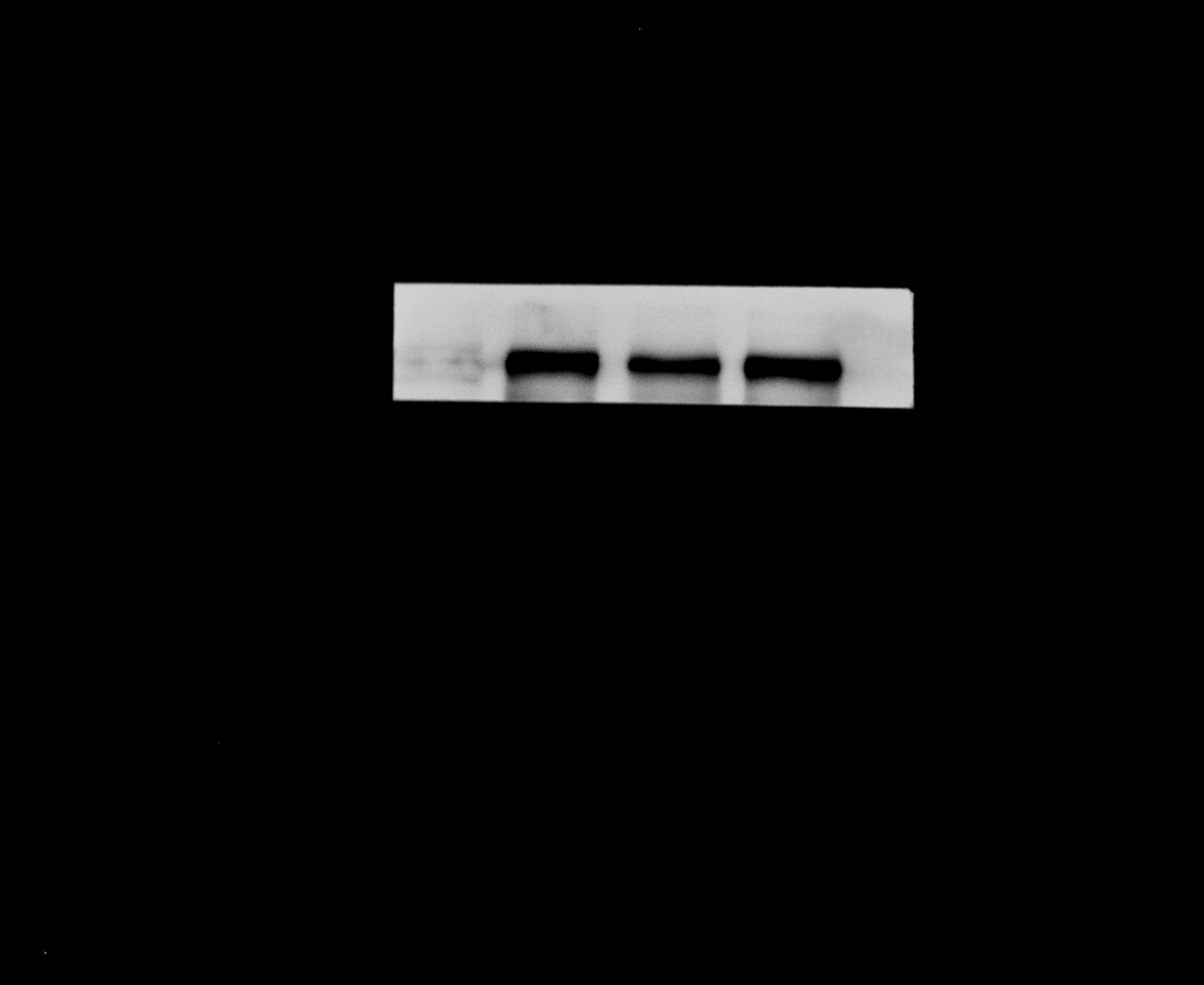


Fig4d C666-1---[（1）si-NC+vector（2）si-GTSE1#1+vector（3）si-GTSE#1+FOXM1]--- β-actin


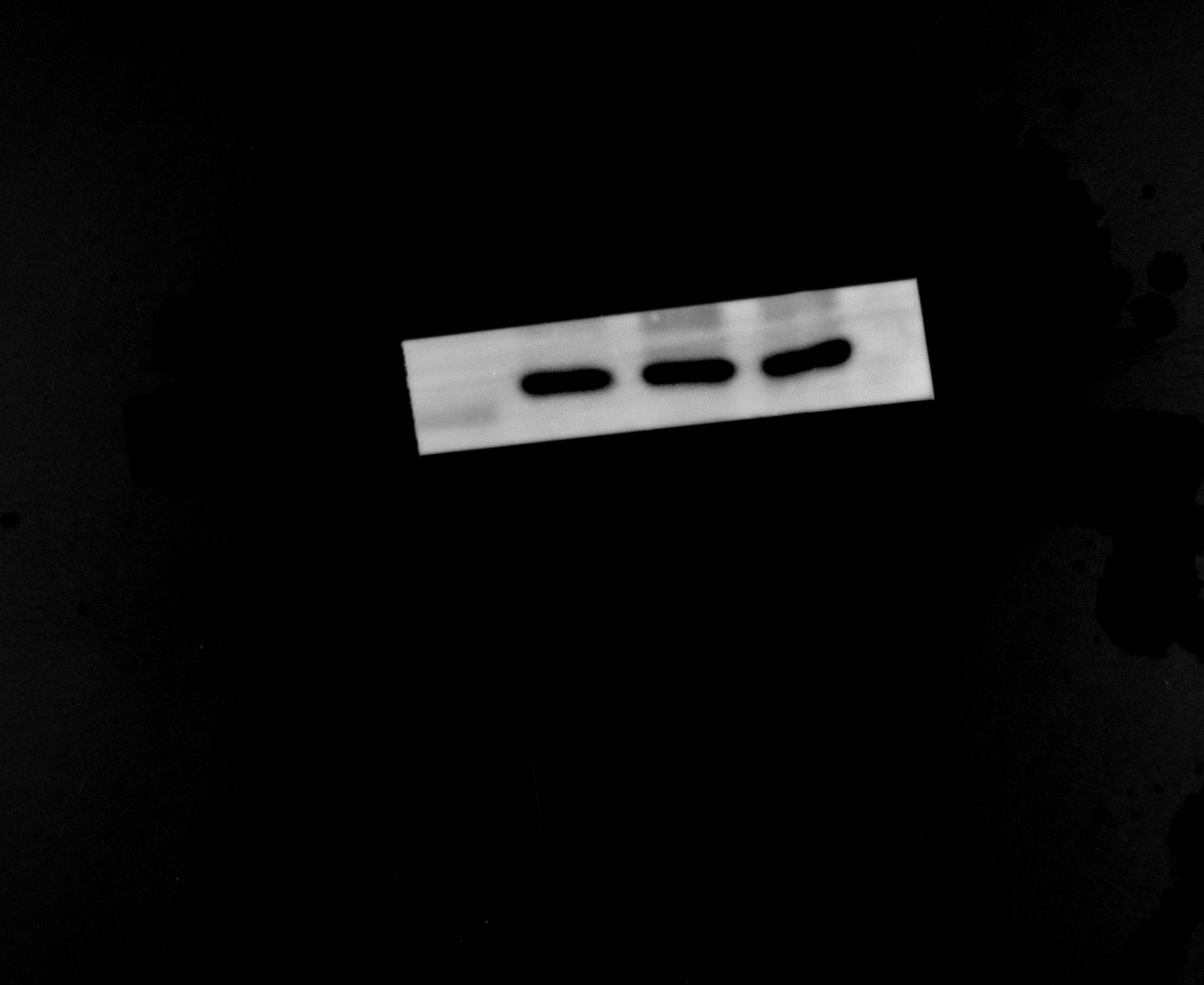


Fig4d SUNE-1---[（1）si-NC+vector（2）si-GTSE1#1+vector（3）si-GTSE#1+FOXM1]--- STMN1


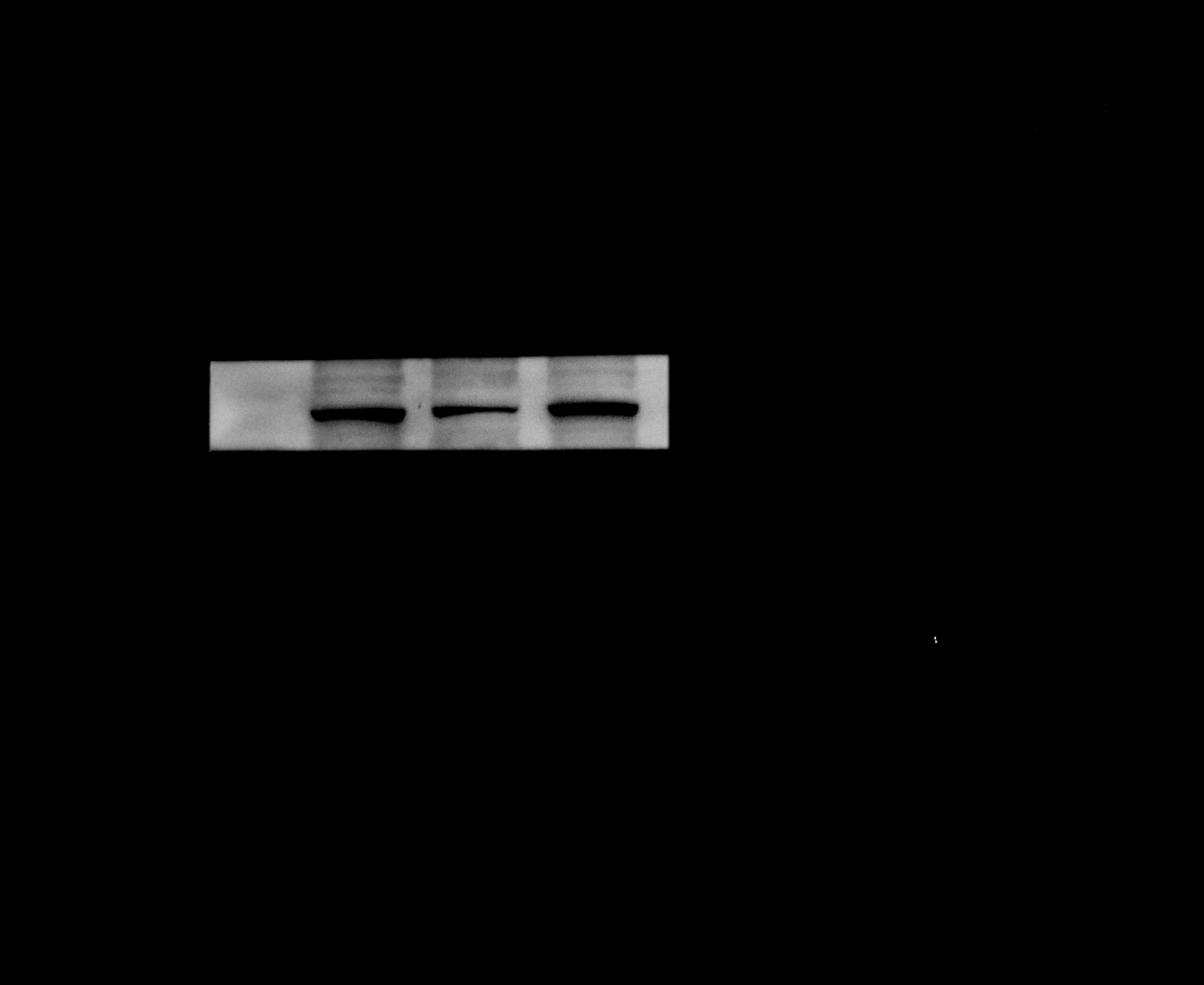


Fig4d SUNE-1---[（1）si-NC+vector（2）si-GTSE1#1+vector（3）si-GTSE#1+FOXM1]--- β-actin


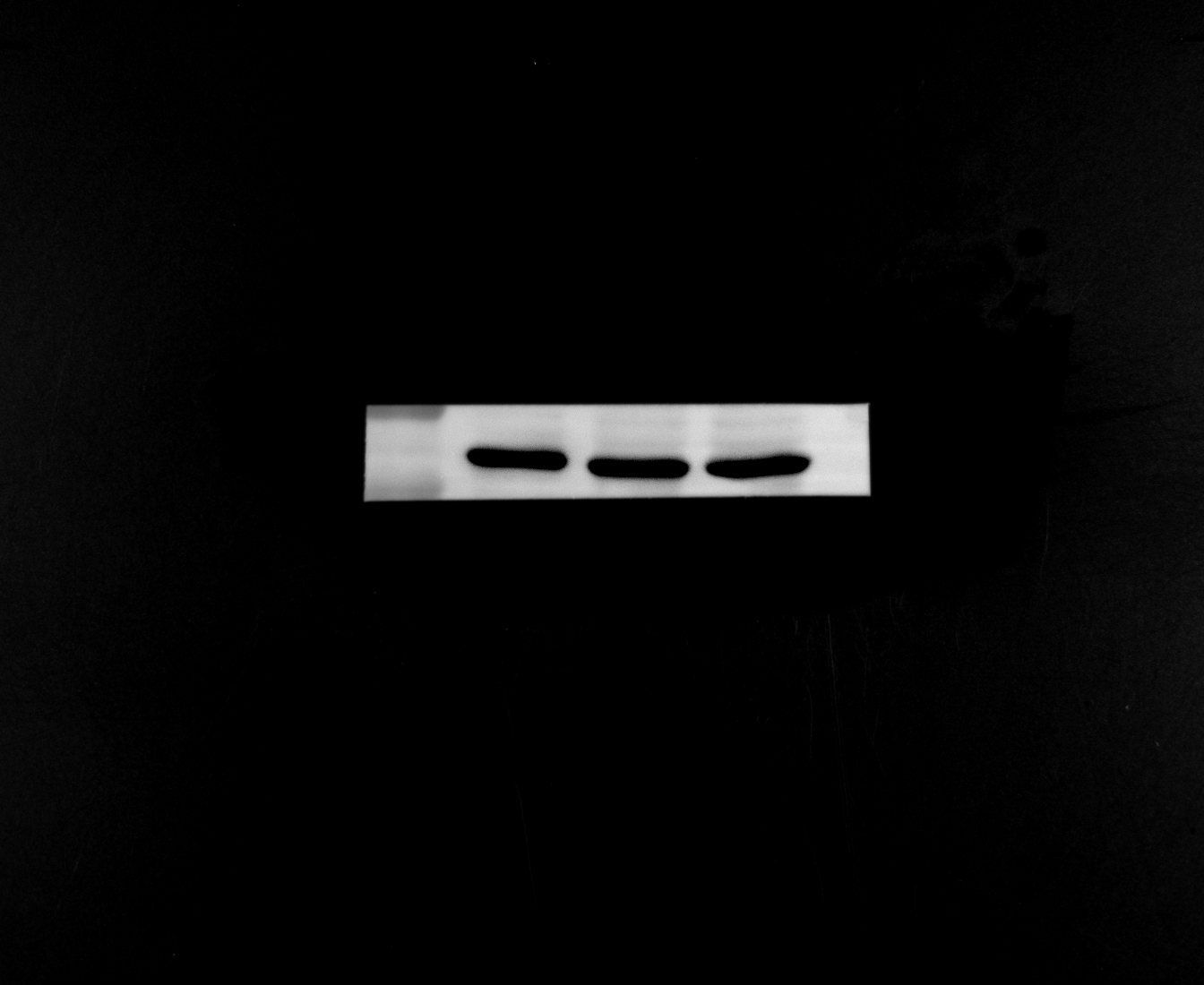

Supplement: Supplementary file 1 — Supplementary Material 1 [file 13008_2024_119_MOESM1_ESM.docx]
